# LPA1-mediated inhibition of CXCR4 attenuates CXCL12-induced signaling and cell migration

**DOI:** 10.1186/s12964-023-01261-7

**Published:** 2023-09-25

**Authors:** Jong Min Hong, Jin-Woo Lee, Dong-Seung Seen, Jae-Yeon Jeong, Won-Ki Huh

**Affiliations:** 1 School of Biological Sciences, Seoul National University, Seoul, 08826 Republic of Korea; 2 GPCR Therapeutics Inc, Gwanak-Gu, Seoul, 08790 Republic of Korea; 3 Institute of Microbiology, Seoul National University, Seoul, 08826 Republic of Korea

**Keywords:** Chemokine receptor 4, Lysophosphatidic acid receptor 1, G protein-coupled receptor, GPCR heteromer, GPCR signaling, Chemotaxis, Cancer, Inflammatory disease

## Abstract

**Background:**

G protein-coupled receptor heteromerization is believed to exert dynamic regulatory impact on signal transduction. CXC chemokine receptor 4 (CXCR4) and its ligand CXCL12, both of which are overexpressed in many cancers, play a pivotal role in metastasis. Likewise, lysophosphatidic acid receptor 1 (LPA_1_) is implicated in cancer cell proliferation and migration. In our preliminary study, we identified LPA_1_ as a prospective CXCR4 interactor. In the present study, we investigated in detail the formation of the CXCR4-LPA_1_ heteromer and characterized the unique molecular features and function of this heteromer.

**Methods:**

We employed bimolecular fluorescence complementation, bioluminescence resonance energy transfer, and proximity ligation assays to demonstrate heteromerization between CXCR4 and LPA_1_. To elucidate the distinctive molecular characteristics and functional implications of the CXCR4-LPA_1_ heteromer, we performed various assays, including cAMP, BRET for G protein activation, β-arrestin recruitment, ligand binding, and transwell migration assays.

**Results:**

We observed that CXCR4 forms heteromers with LPA_1_ in recombinant HEK293A cells and the human breast cancer cell line MDA-MB-231. Coexpression of LPA_1_ with CXCR4 reduced CXCL12-mediated cAMP inhibition, ERK activation, Gα_i/o_ activation, and β-arrestin recruitment, while CXCL12 binding to CXCR4 remained unaffected. In contrast, CXCR4 had no impact on LPA_1_-mediated signaling. The addition of lysophosphatidic acid (LPA) further hindered CXCL12-induced Gα_i/o_ recruitment to CXCR4. LPA or alkyl-OMPT inhibited CXCL12-induced migration in various cancer cells that endogenously express both CXCR4 and LPA_1_. Conversely, CXCL12-induced calcium signaling and migration were increased in *LPAR1* knockout cells, and LPA_1_-selective antagonists enhanced CXCL12-induced Gα_i/o_ signaling and cell migration in the parental MDA-MB-231 cells but not in LPA_1_-deficient cells. Ultimately, complete inhibition of cell migration toward CXCL12 and alkyl-OMPT was only achieved in the presence of both CXCR4 and LPA_1_ antagonists.

**Conclusions:**

The presence and impact of CXCR4-LPA_1_ heteromers on CXCL12-induced signaling and cell migration have been evidenced across various cell lines. This discovery provides crucial insights into a valuable regulatory mechanism of CXCR4 through heteromerization. Moreover, our findings propose a therapeutic potential in combined CXCR4 and LPA_1_ inhibitors for cancer and inflammatory diseases associated with these receptors, simultaneously raising concerns about the use of LPA_1_ antagonists alone for such conditions.

Video Abstract

**Supplementary Information:**

The online version contains supplementary material available at 10.1186/s12964-023-01261-7.

## Background

G protein-coupled receptors (GPCRs) are the largest family of plasma membrane receptors that orchestrate intracellular signaling in response to diverse extracellular stimuli [1, 2]. GPCRs have been extensively studied as drug targets because they are involved in essential physiological processes and pathological conditions and have drug-accessible sites at the cell surface [3]. Approximately 35% of all drugs approved by the Food and Drug Administration (FDA) target GPCRs [3, 4]. Ligand binding induces conformational changes in GPCRs followed by intracellular signaling through activation of heterotrimeric G proteins and recruitment of β-arrestins. GPCR signaling is terminated by phosphorylation of active receptors by GPCR kinases and subsequent binding of β-arrestins to phosphorylated GPCRs [5, 6]. Recent studies suggest that GPCRs can form homomers or heteromeric complexes [7–9]. GPCR heteromerization has been shown to modulate ligand binding, signaling efficacy, and receptor trafficking [10, 11]. For example, heteromerization of sst2A and sst3 somatostatin receptors inhibits sst3 receptor function [12], whereas heteromerization of sst2A receptor and μ-opioid receptor (μOR) cross-phosphorylates and cross-desensitizes each other without affecting the ligand binding and signaling properties of sst2A and μOR [13]. Thus, the identification of novel GPCR heteromers and investigation of heteromer-specific properties will provide new opportunities to understand the mechanism of action of drugs targeting individual GPCRs in the context of GPCR heteromers and to predict potential benefits or complications.

CXC chemokine receptor 4 (CXCR4) is mainly expressed on hematopoietic cells, endothelial cells, and stem cells, and plays an essential role in hematopoietic stem cell homing, leukocyte trafficking, and embryonic development of the hematopoietic, cardiovascular, and nervous systems [14, 15]. CXCR4 is responsible for breast cancer metastasis to organs such as lung, liver, lymph nodes, and bones, which express higher levels of CXCL12 [16]. CXCR4 is overexpressed in a variety of malignancies, including breast, pancreas, thyroid, prostate, kidney, lung, and brain cancers, contributing to tumor growth, angiogenesis, tumor microenvironment interactions, metastasis, and therapeutic resistance [17, 18]. Thus, CXCR4 is regarded as a promising therapeutic target for the treatment of cancer. CXCR4 signals through Gα_i/o_, triggering downstream signaling cascades through phospholipase C (PLC), intracellular calcium mobilization, extracellular signal-regulated kinase (ERK1/2), and protein kinase B (PKB/Akt) [19, 20]. CXCR4 is known to form not only homodimers and homo-oligomers but also heteromers with other chemokine receptors and unrelated receptors. CXCR4-CXCR7 heteromers activate β-arrestin-linked signaling over canonical Gα_i/o_ signaling [21], whereas simultaneous activation of the CXCR4-cannabinoid receptor 2 (CB2) heteromer reduces ERK1/2 phosphorylation, calcium mobilization, and chemotaxis [22].

Lysophosphatidic acid (LPA) is a bioactive phospholipid that regulates cell proliferation, migration, and cytoskeletal reorganization through activation of LPA receptor 1 (LPA_1_) to LPA receptor 6 (LPA_6_) [23]. LPA_1_ is widely expressed in several organs, including the brain, uterus, lung, spleen, and thymus, and has a role in neural development and function, adipogenesis, bone homeostasis, the development of pulmonary fibrosis, and cancer progression [24–26]. LPA_1_ triggers downstream signaling cascades via the mitogen-activated protein kinase (MAPK), PLC, and Rho kinase pathways by signaling through Gα_i/o_, Gα_q/11_, and Gα_12/13_ [27]. LPA_1_ is involved in breast cancer, hepatocellular carcinoma, and pancreatic cancer cell invasion. Heteromerization of LPA_1_ with adhesion GPCR E5 enhances LPA_1_ signaling and metastasis of prostate and thyroid cancers [28, 29]. LPA_1_ also interacts with sphingosine-1-phosphate receptor 1 (S1P_1_) and loosens lymphatic endothelial junctions in lymph nodes to allow efficient lymphocyte trafficking by promoting β-arrestin recruitment to S1P_1_ and inhibiting S1P_1_-mediated Gα_i/o_ signaling [25].

In our preliminary study, we tried to identify novel CXCR4 interactors by screening approximately 160 GPCRs using a high-throughput system for an adenovirus-based bimolecular fluorescence complementation (AdBiFC) assay [30]. LPA_1_ was identified as one of the CXCR4 interactor candidates. In the present study, we investigated in detail the formation of the CXCR4-LPA_1_ heteromer and characterized the unique molecular features and function of this heteromer not only in an ectopic expression system but also in cancer cell lines that endogenously express both receptors. We found that LPA_1_ reduces CXCR4-mediated signaling and cell migration. In contrast, LPA_1_-mediated signaling and β-arrestin recruitment were not affected by CXCR4. Our findings reveal a novel regulatory mechanism for G protein signaling mediated by CXCR4. Furthermore, our data raise concerns about the potential complications in using LPA_1_ antagonists for the treatment of cancer or inflammatory diseases that require the inhibition of both CXCR4 and LPA_1_.

## Methods

### Plasmids

Human *CXCR4*, *LPAR1*, *OPRM1*, *GNA1*, *GNA2*, *GNA3*, *GNAO*, *GNB1*, and *GNG1* cDNAs were obtained from the Missouri S&T cDNA Resource Center and cloned into pENTR201 or pENTR/D-TOPO vectors (Invitrogen) as described previously [30]. If necessary, a stop codon was introduced at the end of the coding sequence using site-directed mutagenesis. *Gaussia* luciferase (Gluc)-PM was a gift from Laszlo Hunyady (#164783, Addgene) [31]. Flag, HA, and Myc tags or Gluc were cloned at the N-terminus of each GPCR using one-step sequence- and ligation-independent cloning (SLIC) [32]. The pcDNA3.1-EYFP and pcDNA3.1-Rluc8 vectors were provided by Hee-Jung Choi at Seoul National University [33]. VN and VC fragments were derived from AdBiFC vectors as described previously [34]. The pcDNA3.1 destination vectors with or without the C-terminal VN, VC, Rluc8, or GFP2 tags were constructed using SLIC. GPCRs were cloned into pcDNA3.1 destination vectors using the Gateway cloning system according to the manufacturer’s instructions (Invitrogen). Plasmids for Gα activation assay (TRUPATH) were a gift from Bryan Roth (#1000000163, Addgene) [35]. Human β-arrestin2 (*ARRB2*) cDNA was obtained from the cDNA Resource Center. Human β-arrestin1 (*ARRB1*) cDNA was cloned by RT-PCR using mRNA from MDA-MB-231 cells, and its sequence was identical to that of *ARRB1* transcript variant 2 (NM_020251). CXCR4-tango plasmid was also a gift from Bryan Roth (#66262, Addgene) [36]. mCitrine_N1 was a gift from Robert Campbell, Michael Davidson, Oliver Griesbeck, and Roger Tsien (#54594, Addgene). To construct pcDNA3.1-mCitrine-β-arrestin1/2, mCitrine was cloned between *Nhe*I and *BamH*I with a C-terminal GGGGSGGGGS linker, and then *ARRB1* and *ARRB2* were cloned between *BamH*I and *EcoR*I using SLIC. For CRISPR-Cas9 gene editing, control guide RNA (sgScramble, 5′-ACGGAGGCTAAGCGTCGCAA-3′) and guide RNAs targeting human *LPAR1* (sg*LPAR1* #1, 5′-AACAGTCAGTCTCCGAGTAT-3′; sg*LPAR1* #2, 5′-CTGTCCACAGTGCGACGTGC-3′) were cloned into lentiCRISPR-v2, a gift from Feng Zhang (#52961, Addgene).

### Cell culture, transfection, and generation of *LPAR1* knockout cells

HEK293A cells were purchased from Invitrogen. HTLA cells were generously provided by Richard Axel at Columbia University. MDA-MB-231, Hs766t, and THP-1 cells were purchased from the American Type Culture Collection (ATCC). MCF7, U937, and Jurkat clone E6-1 cells were obtained from the Korean Cell Line Bank (KCLB). 8505C cells were obtained from the Japanese Collection of Research Bioresources (JCRB) cell bank. HEK293A, Hs766t, and HTLA cells were cultured in Dulbecco’s modified Eagle’s medium (HyClone) supplemented with 10% fetal bovine serum (FBS; HyClone). For HTLA cells, puromycin (1 μg/ml) and hygromycin (100 μg/ml) were additionally supplemented. 8505C cells were cultured in Minimum Essential Medium Eagle (Sigma-Aldrich) supplemented with 10% FBS. MDA-MB-231, MCF7, Jurkat, U937, and THP-1 cells were cultured in Roswell Park Memorial Institute (RPMI) 1640 medium (HyClone) supplemented with 10% FBS. All cells were cultured at 37°C in a 5% CO_2_ humidified atmosphere. For transient transfection, HEK293A cells were seeded at a density of 5 × 10^5^ cells per well in 6-well plates or 2 × 10^5^ cells per well in 12-well plates. Transient transfections were performed using PEI MAX (1 mg/ml; molecular weight 40,000; Polysciences) diluted in Opti-MEM (Gibco) with a DNA:PEI MAX ratio of 1:4. *LPAR1* gene and control knockout MDA-MB-231 cells were generated by transducing cells with lentiCRISPR-sg*LPAR1* #1, lentiCRISPR-sg*LPAR1* #2, or lentiCRISPR-sgScramble lentiviruses. Knockout cells were selected with 2 μg/ml puromycin (InvivoGen). To assess the mutation of *LPAR1* target #1, PCR was conducted using the primers 5’-TTATAACCGAAGTGGAAAGCA-3’ and 5’-AGATGTGAGCATAGAGAACC-3’. The resulting PCR amplicon was digested with *Ppu*MI (NEB) and the subjected to Sanger sequencing to confirm the knockout of *LPAR1*. For *LPAR1* target #2, PCR was performed using the primers 5’-TTGCTTGATTTTAGTAACGTCC-3’ and 5’-AGGGGGAGGCTGTTTATCCT-3’. The resulting PCR amplicon was digested with *Cac*8I (NEB) and then subjected to Sanger sequencing.

### Reagents and antibodies

CXCL12 was purchased from Peprotech. LPA (1-oleoyl lysophosphatidic acid) was purchased from Biogems. Alkyl-OMPT (D-sn-1-O-oleyl-2-O-methyl-glyceryl-3-phosphothionate) was purchased from Echelon Biosciences. Burixafor and TZ14011-AF488 were provided by GPCR Therapeutics Inc. AM095, AM966, BMS986020, and forskolin were purchased from MedChemExpress. Ro6842262 was purchased from Tocris Bioscience. Coelenterazine h, coelenterazine 400a, and coelenterazine native were purchased from Nanolight Technology. Alexa Fluor 568-conjugated goat anti-mouse IgG and Alexa Fluor 568-conjugated goat anti-rabbit IgG were purchased from Invitrogen. Anti-ERK1/2 (#9102), anti-phospho-ERK1/2 (T202/Y204) (#4370), anti-Myc tag (#2276), and anti-HA tag (#3724) antibodies were purchased from Cell Signaling Technology. Anti-Flag antibody clone 2H8 (#KAL-KO602) was purchased from Cosmo Bio Co. Anti-ERK1/2 (#sc-94), anti-β-Actin-HRP (#sc-47778 HRP), and anti-CXCR4 (4G10, #sc-53434) antibodies were purchased from Santa Cruz Biotechnology. Mouse IgG control antibody (#I-2000) was purchased from Vector Laboratories. Anti-mouse APC antibody (#F0101B) and anti-LPA_1_ antibody (#MAB99631) were purchased from R&D Systems. Anti-rabbit IgG HRP-conjugated antibody (#A9169) was purchased from Sigma-Aldrich. M1/M2 probe-conjugated anti-CXCR4 (mAb1) and M2 probe-conjugated IgG isotype antibodies were provided by GPCR Therapeutics Inc.

### Immunofluorescence staining and bimolecular fluorescence complementation (BiFC) assay

**Fig. 1:**
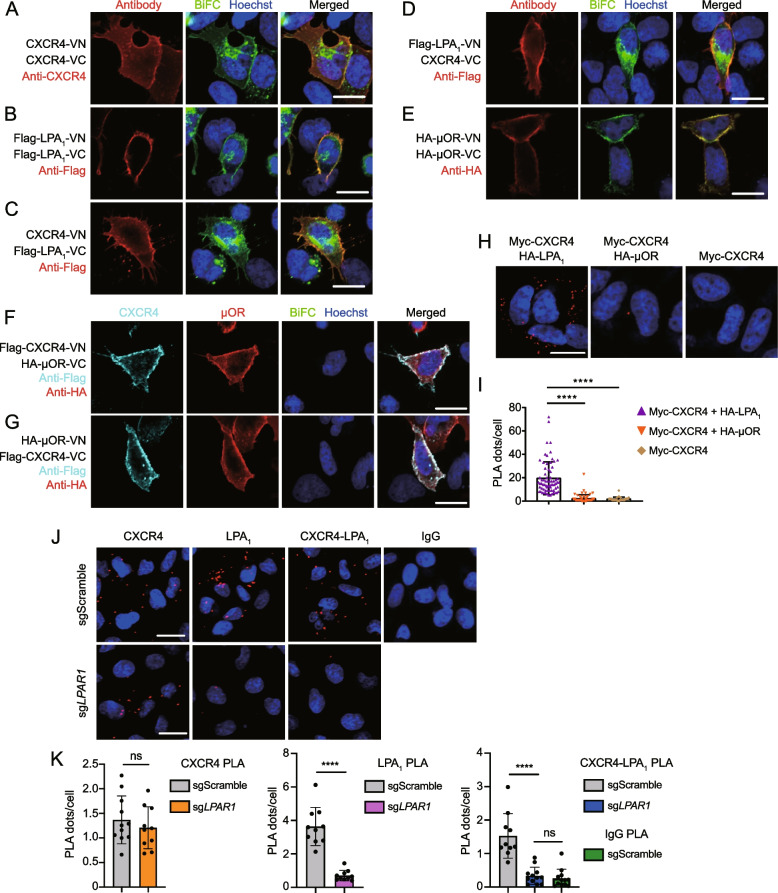
CXCR4 comes in close physical proximity to LPA_1_. **A**-**G** Representative confocal images to detect GPCR interactions in HEK293A cells using the BiFC assay. Cells were transfected with CXCR4-VN and CXCR4-VC (**A**), Flag-LPA_1_-VN and Flag-LPA_1_-VC (**B**), CXCR4-VN and Flag-LPA_1_-VC (**C**), Flag-LPA_1_-VN and CXCR4-VC (**D**), HA-μOR-VN and HA-μOR-VC (**E**), Flag-CXCR4-VN and HA-μOR-VC (**F**), or HA-μOR-VN and Flag-CXCR4-VC (**G**). Cell surface expression of CXCR4, LPA_1_, and μOR was visualized by staining cells with anti-CXCR4, anti-Flag, or anti-HA antibodies without permeabilization. Nuclei were stained with Hoechst 33342. **H** PLA was performed to visualize CXCR4 and LPA_1_ heteromerization in HEK293A cells. Cells transiently expressing Myc-CXCR4 and HA-LPA_1_, Myc-CXCR4 and HA-μOR, or Myc-CXCR4 alone were first stained with mouse anti-CXCR4 and rabbit anti-HA antibodies and then with anti-mouse and anti-rabbit secondary antibodies conjugated with plus and minus PLA probes. **I** The number of PLA dots per cell was counted using ImageJ software. Images containing a total of positive 30 to 90 cells were analyzed. Data represent the mean ± SEM of *n* = 3 independent experiments. Statistical significance was tested using unpaired two-tailed Student’s *t* test. ****P < 0.0001. **J** PLA was performed to visualize CXCR4 and LPA_1_ heteromerization in MDA-MB-231 cells. Representative PLA images for the detection of CXCR4, LPA_1_, and CXCR4-LPA_1_ heteromers using M1/M2 probe-conjugated anti-CXCR4 and mouse anti-LPA_1_ antibodies are shown. IgG isotype antibody was used for control experiments. **K** The number of PLA dots per cell was quantified using ImageJ software. For each independent experiment, 3 to 4 field images were used for quantification. Data represent the mean ± SEM of *n* = 3 independent experiments. Statistical significance was tested using unpaired two-tailed Student’s *t* test. ****P < 0.0001; ns, not significant. Scale bars: 20 μm

HEK293A cells were plated in a 12-well plate and transfected with GPCR-VN and GPCR-VC at a ratio of 1:1 using PEI MAX. The next day, cells were dissociated with Accutase and plated on a 96-well clear-bottom black plate (#655090, Greiner). The next day, cells were fixed with 4% paraformaldehyde for 15 min at room temperature and stained first with anti-CXCR4 (1:200), anti-Flag (1:2000), or anti-HA (1:2000) antibodies and then with Alexa Fluor 568-conjugated goat anti-mouse IgG (1:1000) or goat anti-rabbit IgG (1:1000) antibodies. For Fig. 1F and G, anti-mouse APC-conjugated secondary antibody was used for staining anti-Flag antibody. To examine colocalization of Rluc8-tagged CXCR4 or LPA_1_ and GFP2-tagged CXCR4 or LPA_1_, cells were fixed with 4% paraformaldehyde for 15 min at room temperature and permeabilized with 0.1% Triton X-100 for 15 min at room temperature. Cells were blocked using 0.5% BSA for 1 h at room temperature and stained with anti-HA (1:2000) antibody and then with Alexa Fluor 568-conjugated goat anti-rabbit IgG (1:1000) antibody. Hoechst 33342 was used for nuclear staining. BiFC and immunofluorescence images were acquired with an LSM 700 laser scanning confocal microscope (Zeiss) using a 20 × objective. Hoechst 33342 was excited by a 405 nm excitation laser, and the emission signal was acquired by 410 to 480 nm emission filter sets. Venus and GFP were excited by a 480 nm excitation laser, and the emission signal was acquired by 490 to 540 nm emission filter sets. Alexa Fluor 568 was excited by a 488 nm excitation laser, and the emission signal was acquired by 570 to 620 nm emission filter sets. APC was excited by a 639 nm excitation laser and detected through a 640 to 680 nm emission filter sets.

### Proximity ligation assay (PLA)

PLA was performed using a NaveniFlex MR kit according to the manufacturer’s instructions (Navinci). In brief, transfected HEK293A or endogenous MDA-MB-231 cells were seeded in poly-D-lysine-coated 96-well clear-bottom black plates (#655090, Greiner). The next day, cells were fixed with 4% paraformaldehyde for 15 min at room temperature, blocked with a blocking solution. HEK293A cells were stained with mouse anti-CXCR4 (1:2000) and rabbit anti-HA tag (1:15,000) antibodies at 4°C overnight. MDA-MB-231 cells were stained with M1/M2 probe-conjugated anti-CXCR4 (10 μg/ml), mouse anti-LPA_1_ (1:500), or M2 probe-conjugated IgG isotype (1 μg/ml) antibodies at 37 °C for 60 min. After washing, the samples were incubated with secondary anti-mouse and anti-rabbit antibodies conjugated with plus and minus PLA probes and processed for ligation and amplification in the presence of Texas Red. After nuclear staining with Hoechst 33342, Z-stack images were acquired using an LSM 700 laser scanning confocal microscope (Zeiss) with a 20 × objective for quantification and a 20 × or 40 × objective for representative images. PLA dots were quantified using ImageJ software (National Institutes of Health). Hoechst-stained nuclei were counted and PLA dots of each cell were calculated using the “Analyze Particles” command.

### Analysis of cell surface expression of GPCRs

For ELISA experiments, cells were transfected with Flag-tagged GPCRs in 6-well plates. One day after transfection, cells were dissociated with Accutase and seeded in poly-D-lysine-coated 96-well plates. 1% BSA was used as a blocking agent. Cells were incubated with anti-Flag antibody (1:4000) at 4°C overnight and then with anti-mouse HRP-conjugated antibody (1:50,000) in the dark for 1 h. Cells were washed three times with 1% BSA buffer before the ELISA absorbance was measured using an EnVision Multilabel Plate Reader (Perkin Elmer). ELISA absorbance values were measured at 450 nm wavelength and were averaged and normalized by subtracting the value for the nontransfected cells. For flow cytometry experiments, HEK293A or MDA-MB-231 cells were dissociated with Accutase, and stained with anti-Flag, anti-Myc, anti-CXCR4 (4G10) mouse monoclonal antibodies, or control mouse IgG for 1 h on ice. After labeling with APC-conjugated anti-mouse IgG antibody, cells were analyzed using a BD FACSCanto II flow cytometer (BD Bioscience). Flow cytometry data were analyzed with FlowJo software, and the mean fluorescence intensity was used to compare the relative cell surface expression of GPCRs.

### GloSensor cAMP assay

The production of cAMP was measured using a GloSensor cAMP assay (Promega). HEK293A cells were plated in 12-well or 6-well plates and transfected with pGloSensor-22F, pcDNA3-myc-CXCR4, pcDNA3-Flag-LPA_1_, and pcDNA3-HA-μOR plasmid. The next day, cells were dissociated with DPBS containing 2 mM EDTA and plated in 96-well white plates. Two days after transfection, cells were washed with CO_2_-independent medium (Gibco) containing 0.1% BSA and incubated in the dark with 2 mM D-luciferin (BioVision) for 90 min at room temperature. Antagonists were administered 30 min prior to agonist treatment. To measure ligand-induced inhibition of cAMP production, cells were first stimulated with 3 μM forskolin followed by agonist treatment. Luminescence was measured for 40 min with an integration time of 1 s per well using an LB942 Mithras microplate reader and luminescence obtained between 5 and 20 min was averaged for calculation. To measure the production of cAMP in MDA-MB-231 cells, the GloSensor-22F coding sequence was amplified using PCR, cloned into pENTR/D-TOPO, and then subcloned into pLenti-X1-Hygro-DEST (694–6) using Gateway cloning (Invitrogen). pLenti-X1-Puro-DEST (694–6) was a gift from Eric Campeau & Paul Kaufman (#17297, Addgene) and was used to change the antibiotic marker gene from puromycin to hygromycin. MDA-MB-231 cells were transduced with lentiviruses encoding Glosensor-22F, and stable cells were selected with hygromycin (400 μg/ml; InvivoGen). MDA-MB-231-22F cells were plated in 96-well plates, and cAMP production was measured as described above.

### Western blotting

HEK293A cells were seeded in 12-well plates one day before transfection with pcDNA3-Myc-CXCR4 or pcDNA3-Flag-LPA_1_ alone, or in combination. Two days after transfection, HEK293A cells were serum-starved in DMEM containing 0.1% BSA for 4 h and then treated with agonists. Cells were washed with ice-cold PBS and lysed with lysis buffer (50 mM Tris–Cl, pH 7.4, 150 mM NaCl, 5 mM EDTA, 1 mM EGTA, and 1% NP-40) supplemented with 1 μg/ml leupeptin, 1 μg/ml pepstatin A, 1 mM benzamidine, 1 mM PMSF, 10 mM NaF, 1 mM β-glycerophosphate, 1 mM sodium orthovanadate, and 1 mM sodium pyrophosphate. Cell lysates were incubated for 30 min on ice and protein concentrations of the supernatants were determined by Bradford or BCA protein assay. SDS-PAGE analysis was performed with 10% separating gels and transferred to nitrocellulose membranes. Immunoblotting was performed with anti-ERK1/2 (1:2000), anti-phospho-ERK1/2 (1:1000), or anti-β-actin HRP-conjugated (1:10,000) antibodies in 5% skim milk in TBS containing 0.1% Tween 20. After washing the membranes, blots were incubated with HRP-conjugated goat anti-rabbit (1:5000) antibody. Immunoreactivity was detected by enhanced chemiluminescence using EZ-Capture II (Atto Technology), and data quantification was performed using ImageJ software.

### Bioluminescence resonance energy transfer (BRET) saturation assay

A fixed amount of HA-CXCR4-Rluc8 or Flag-LPA_1_-Rluc8 plasmid (each 15 ng) and increasing amounts of Flag-LPA_1_-GFP2 or CXCR4-GFP2 plasmid (0 to 390 ng), respectively, were used for transfection. As a control, a fixed amount of HA-μOR-Rluc8 plasmid (40 ng) and increasing amounts of CXCR4-GFP2 plasmid (0 to 390 ng) were used for transfection. Two days after transfection, cells were dissociated using DPBS containing 2 mM EDTA, pelleted by centrifugation at 500*g* for 3 min, and resuspended in HBSS (HyClone) containing 0.1% BSA. Cells were counted and 60,000 cells were dispensed per well into a 96-well white plate (#3917, Corning). Coelenterazine 400a (Nanolight Technology) was added to a final concentration of 5 μM. BRET2 signals were read in an LB942 Mithras microplate reader (TriStar2 LB 942, Berthold Technologies) using a 410 nm filter for Rluc8 and a 510 nm filter for GFP2 with an integration time of 0.5 s per well. For fluorescence measurement, cells were seeded in a 96-well black plate (#3340, Corning), and signals were read in an LB942 Mithras microplate reader using a 480 nm excitation filter and a 540 nm emission filter with an integration time of 0.3 s per well. The BRET2 ratio was calculated by dividing the GFP2 signal by the Rluc8 signal. The net BRET ratio was calculated by subtracting the BRET ratio obtained from cells expressing Rluc8 alone from the BRET ratio obtained from cells expressing Rluc8 and GFP2.

### G protein activation assay

Gα_i/o_ activation was quantified by determining the ligand-induced BRET change between Gα and Gβγ subunits. HEK293A cells were seeded in 6-well plates. For BRET1 (Additional file 1: Fig. S3A-C), Myc-CXCR4 (30 ng) was transfected with each 100 ng of Gα_i1_-Rluc8, Gα_i2_-Rluc8, Gα_i3_-Rluc8, or Gα_oA_-Rluc8, Gβ_1_, and EYFP-Gγ_1_ in the presence or absence of Flag-LPA_1_ (120 ng). For BRET2 using TRUPATH plasmids (Fig. 3B, C, Additional file 1: Fig. S3D-J), Flag-LPA_1_ was transfected in combination with each 100 ng of Gα_i1_-Rluc8, Gα_i2_-Rluc8, Gα_i3_-Rluc8, or Gα_oA_-Rluc8, Gβ_3_, and GFP2-Gγ_8_, or GFP2-Gγ_9_ in the presence or absence of Myc-CXCR4 as described previously [35]. For Gα_i_ recruitment to GPCR, HEK293A cells were transfected with CXCR4-mCitrine or Flag-LPA_1_-mCitrine, Gα_i1_-Rluc8, Gβ_1_, and Gγ_1_ in the presence or absence of Flag-LPA_1_ or Myc-CXCR4, respectively. One day after transfection, cells were dissociated using DPBS containing 2 mM EDTA and centrifuged at 500*g* for 3 min. Cells were resuspended in DMEM without phenol red (Gibco) containing 5% FBS and seeded into a 96-well white plate at 5 × 10^4^ per well in 100 μl. The next day, cells were washed with HBSS containing 0.1% BSA, and antagonists were administered for 30 min before agonist treatment. Cells were treated with coelenterazine h for BRET1 and coelenterazine 400a for BRET2 to a final concentration of 5 μM. The plates were incubated for 5 min at room temperature in the dark and treated with multiple doses of agonist in triplicate. Plates were read in an LB942 Mithras microplate reader with a 480 nm filter for Rluc8 and a 540 nm filter for EYFP to measure BRET1 and a 410 nm filter for Rluc8 and a 510 nm filter for GFP2 to measure BRET2 at an integration time of 0.1 s per well for 5 min. The BRET1 ratio was calculated by dividing the EYFP signal by the Rluc8 signal. The BRET2 ratio was calculated by dividing the GFP2 signal by the Rluc8 signal. The ΔBRET ratio was calculated by subtracting the vehicle BRET ratio from the ligand-induced BRET ratio.

### β-Arrestin recruitment assay using BRET

HEK293A cells were transfected with CXCR4-Rluc8 or Flag-LPA_1_-Rluc8, mCitrine-β-arrestin1 (300 ng), and mCitrine-β-arrestin2 (300 ng) in the presence or absence of Flag-LPA_1_ or Myc-CXCR4, respectively. For β-arrestin1/2 saturation assay, we transfected CXCR4-Rluc8 (20 ng) and mCitrine-β-arrestin1/2 (0 to 400 ng) in the presence or absence of Flag-LPA_1_ (100 ng). One day after transfection, cells were dissociated with DPBS containing 2 mM EDTA and centrifuged at 500*g* for 3 min. Cells were resuspended in DMEM without phenol red containing 5% FBS and seeded in a 96-well white plate at 5 × 10^4^ per well in 100 μl. The next day, cells were washed with HBSS in 0.1% BSA and incubated for 30 min to reach equilibrium at 37°C in a 5% CO_2_ humidified atmosphere. Coelenterazine h was added to a final concentration of 5 μM and incubated for 5 min at room temperature in the dark before ligand stimulation. After ligand application, the plates were placed in an LB942 Mithras microplate reader with a 480 nm filter for Rluc8 and a 540 nm filter for mCitrine. Each plate was measured for 15 min. For BRET saturation assay, the plates were measured for 30 min. For fluorescence measurement, cells were seeded one day before measurement in a 96-well black plate (#655090, Greiner), and signals were read in an LB942 Mithras microplate reader using a 480 nm excitation filter and a 540 nm emission filter with an integration time of 0.3 s per well. The BRET ratio was calculated by dividing the mCitrine signal by the Rluc8 signal. In each condition, the ΔBRET ratio was calculated by subtracting the basal BRET ratio observed in cells expressing only CXCR4-Rluc8.

### β-Arrestin recruitment using TANGO assay

For TANGO assay, the v2 tail in the CXCR4-tango plasmid was removed to observe β-arrestin2 recruitment more specifically. HTLA cells were seeded in a 12-well plate at a density of 4 × 10^5^ per well, and then transfected with CXCR4-tango (50 ng) and pcDNA3-Rluc8 (10 ng) in the presence or absence of HA-LPA_1_ (250 ng). After 24 h of transfection, cells were serum starved for at least 4 h, and then stimulated with CXCL12 overnight. The next day, luciferase activities were measured according to the manufacturer’s protocols of the Dual Luciferase Kit (Promega).

### Ligand binding assay using Gluc BRET

Ligand binding assay was performed as previously described [31]. HEK293A cells were transfected with Gluc-CXCR4 alone or in combination with Flag-LPA_1_. One day after transfection, cells were dissociated with Accutase (StemCell Technologies) and centrifuged at 500*g* for 3 min. Cells were resuspended in DMEM without phenol red containing 5% FBS and seeded in a 96-well white plate at 2 × 10^4^ per well. The next day, cells were washed with HBSS and serum-starved for 2 h. In the saturation binding experiments, TZ14011-AF488 was treated in the presence or absence of 20 μM IT1t and incubated for 30 min at room temperature until reaching an equilibrium state. In the competition experiments, the medium was removed from each well, and 40 μl of TZ14011-AF488 was added, followed by a 30-min incubation at room temperature. Next, 10 μl of Coelenterazine native was added to achieve a final concentration of 5 μM, and the mixture was incubated for 5 min. Competing unlabeled CXCL12 (10 μl) was then manually added. Each plate was read using an LB942 Mithras microplate reader with a 480 nm filter for Gluc and a 540 nm filter for Alexa Fluor 488. Luminescence was measured at an integration time of 0.1 s per well for a duration of 30 min. The BRET ratio was calculated by dividing the Alexa Fluor 488 signal by the Gluc signal. The specific BRET ratio was calculated by subtracting the nonspecific BRET ratio from the TZ14011-AF488-induced BRET ratio. The ΔBRET ratio was calculated by subtracting the vehicle BRET ratio from the fluorescent ligand-induced BRET ratio.

### Calcium flux assay

MDA-MB-231 cells were plated in 96-well black clear-bottom plates (#655090, Greiner). Cells were washed with assay buffer (HBSS without phenol red and with 20 mM HEPES and 0.1% BSA) and loaded with Cal-520AM (5 μM; AAT Bioquest) for 2 h at 37°C. Cells were washed twice with assay buffer and incubated at 37°C for an additional 20 min. Plates were loaded into a FlexStation 3 microplate reader (Molecular Devices), and ligands were injected. Intracellular calcium mobilization was measured for 130 s at 37°C with an excitation of 485 nm and an emission of 525 nm. The area under curve was calculated by GraphPad Prism 7 software.

### Transwell migration assay

MDA-MB-231 cells were serum-starved overnight with RPMI 1640 containing 0.1% BSA and dissociated with Accutase. Cells were resuspended in RPMI 1640 containing 0.1% BSA and loaded into the upper chamber of a Boyden chamber plate (8 μm pore size; Costar) precoated with collagen I (50 μg/ml; Advanced BioMatrix). Antagonists were added to the cells 30 min before plating the cells in the upper chamber. The upper chambers were placed in plates containing antagonists and chemoattractants in the lower chambers. After incubation at 37°C for 3 h, the non-migrated cells in the upper chamber were gently removed with a cotton swab. The migrated cells in the lower part of the upper chamber were soaked in 4% formaldehyde for 15 min at room temperature. After washing once with DPBS, the migrated cells were stained with 0.05% crystal violet (#61135, Sigma-Aldrich) for 30 min. Images were taken under an Axio Observer Z1 light microscope with a 10 × objective (Zeiss), and migrated cells were manually counted using ImageJ software. For ligand-induced cross-inhibition of chemotaxis, adherent cells (HEK293A, MDA-MB-231, 8505C, MCF7, and Hs766t) were serum-starved overnight with media containing 0.1% BSA and then dissociated with Accutase. Suspension cells (Jurkat, U937, and THP-1) were serum-starved overnight with media containing 0.1% BSA prior to the migration assay. Cells were loaded into the upper chamber, and CXCL12, alkyl-OMPT, or LPA was added to the upper chamber to test cross-inhibition. LPA, alkyl-OMPT, or CXCL12 was loaded into the lower chamber to induce chemotaxis. For adherent cells, the upper chamber (8 μm pore size; Costar or SPL Life Science) was precoated with collagen I. For suspension cells, Boyden chamber plates with 5 μm pore size inserts (Costar) were used. After staining with crystal violet, images were analyzed to evaluate adherent cell migration. The migration of suspension cells was evaluated by counting migrated cells for 1 min using a BD FACSCanto II flow cytometer with a sample flow rate of 2 μl/sec.

### Quantitative real-time PCR

Total RNA was extracted using the RNeasy mini kit (Qiagen), and DNA was removed using DNase I (#AMPD1-1KT, Sigma-Aldrich). cDNA was synthesized using the ReverTra Ace qPCR RT kit (#FSQ-101, Toyobo). Quantitative PCR was performed with the SensiFAST SYBR Lo-ROX kit (#BIO-94005, Bioline) using the QuantStudio 3 Real-Time PCR system (Thermo Fisher Scientific). GAPDH was used as the reference gene for normalization and mRNA abundance was quantified using the threshold cycle method. The primer sequences used are: CXCR4: 5′-CCACCATCTACTCCATCATCTTC-3′ and 5′-ACTTGTCCGTCATGCTTCTC-3′; LPAR1: 5′-CGCCAGAGGACTATGAGAATG-3′ and 5′-CAGGAGTCCAGCAGATGATAAA-3′; LPAR2: 5′-TTGTCATCATCCTGGGGGCG-3′ and 5′-GCCTCGGCCAACAGTAGGAA-3′; LPAR3: 5′-GTCTTAGGGGCGTTTGTGGT-3′ and 5′-GTTCACGACGGAGTTGAGCA-3′; LPAR4: 5′-AGTGTGGATCGTTTCCTGGC-3′ and 5′-GCCTTCAAAGCAGGTGGTGG-3′; LPAR5: 5′-CTCGCGCAATCCGAAAGGTC-3′ and 5′-GCATGTGTGTTCAGAGGGCG-3′; LPAR6: 5′-AGCATGGTGTTTGTGCTTGGG-3′ and 5′-TGGCCAATTCCGTGTTGTGAA-3′; and GAPDH: 5′-ATGACATCAAGAAGGTGGTGAA-3′ and 5′-GCTGTTGAAGTCAGAGGAGAC-3′.

### Bioinformatics analysis of The Cancer Genome Atlas (TCGA) datasets

Gene expression profiling for breast invasive carcinoma, pancreatic adenocarcinoma, thyroid carcinoma, and acute myeloid leukemia patients was obtained from TCGA. Overall survival (OS) was evaluated by the Kaplan–Meier method. Expression and clinical data of TCGA patients were downloaded using Xenabrowser (https://xenabrowser.net/). Both violin plots for expression data and Kaplan–Meier survival curves were constructed using R version 4.1.2 (http://www.r-project.org/).

### Statistical analysis

Data are expressed as the mean ± SEM or mean ± SD from *n* independent experiments that were performed on different days. Data were analyzed using GraphPad Prism 7 software. Statistical analysis was performed as indicated in the figure legends.

## Results

### CXCR4 and LPA_1_ form heteromers

In our preliminary study, LPA_1_ was identified as a CXCR4 interactor candidate through a high-throughput BiFC-based screen in U2-OS cells cotransduced with adenoviral vectors encoding GPCRx-VN (the N-terminal fragment of Venus) and GPCRy-VC (the C-terminal fragment of Venus) (data not shown). To confirm the previous results, the interaction between CXCR4 and LPA_1_ was assessed in HEK293A cells using a BiFC assay. To examine the surface expression of each GPCR, transfected cells were stained with anti-CXCR4, anti-Flag, or anti-HA antibody without permeabilization (Fig. 1A-G). Reconstituted Venus signals were detected in cells coexpressing CXCR4-VN and CXCR4-VC (Fig. 1A), LPA_1_-VN and LPA_1_-VC (Fig. 1B), and μOR-VN and μOR-VC (Fig. 1E), demonstrating the presence of CXCR4, LPA_1_, and μOR homomers in these cells. When cells were transfected with CXCR4-VN and LPA_1_-VC or CXCR4-VC and LPA_1_-VN, BiFC signals were detected at both the plasma membrane and cytoplasm (Fig. 1C, D), suggesting the occurrence of heteromerization between CXCR4 and LPA_1_. In contrast, the BiFC signal was not observed in cells transfected with CXCR4-VN and μOR-VC (Fig. 1F) or CXCR4-VC and μOR-VN (Fig. 1G), even though CXCR4-VN or CXCR4-VC was expressed adequately and colocalized with μOR at the cell surface. This observation is consistent with a previous report indicating the lack of physical interaction between CXCR4 and μOR [37].

To further validate the interaction between CXCR4 and LPA_1_, we first performed immunofluorescence staining to check for the expression of CXCR4 and LPA_1_ at the same localization. We transfected HA-CXCR4-Rluc8 and Flag-LPA_1_-GFP2 or HA-LPA_1_-Rluc8 and CXCR4-GFP2 constructs into HEK293A cells. After fixing and permeabilization, the cells were stained with rabbit anti-HA monoclonal antibody. GFP2 was visualized through GFP green fluorescence. As shown in Additional file 1: Fig. S1A, B, CXCR4 and LPA_1_ were effectively expressed and colocalized on the cell membrane. We next performed BRET saturation experiments for homomerization and heteromerization of CXCR4 and LPA_1_ in HEK293A cells. Increasing the amounts of CXCR4-GFP2 or LPA_1_-GFP2 together with a constant amount of CXCR4-Rluc8 or LPA_1_-Rluc8, respectively, resulted in hyperbolic increases in the BRET ratio (Additional file 1: Fig. S1C, D). Increasing the amounts of LPA_1_-GFP2 together with a constant amount of CXCR4-Rluc8 or vice versa also resulted in hyperbolic increases in the BRET ratio (Additional file 1: Fig. S1E). The BRET_50_ values for CXCR4-LPA_1_ heteromers were higher than those for CXCR4 homomers but lower than those for LPA_1_ homomers (Table 1). These results suggest that CXCR4 homomers are formed with the highest efficiency, while CXCR4-LPA_1_ heteromers are formed more efficiently than LPA_1_ homomers in cells. In contrast, HEK293A cells expressing a constant amount of μOR-Rluc8 and increasing amounts of CXCR4-GFP2 exhibited an almost linear increase in the BRET ratio, with low BRET_Max_ and high BRET_50_ values, indicating that CXCR4 and μOR have little interaction. The expression levels of RLuc8-tagged GPCRs remained largely unperturbed during BRET saturation experiments (Additional file 1: Fig. S1F-J).

**Table 1:** BRET_Max_ and BRET_50_ values from the BRET saturation assay

	HA-CXCR4-Rluc8: CXCR4-GFP2	HA-LPA_1_-Rluc8: Flag-LPA_1_-GFP2	HA-CXCR4-Rluc8: Flag-LPA_1_-GFP2	HA-LPA_1_-Rluc8: CXCR4-GFP2	HA-μOR-Rluc8: CXCR4-GFP2
BRET_Max_	1.140	0.599	0.426	0.398	0.044
BRET_50_	0.244	0.629	0.354	0.450	1.943

To rule out possible nonspecific interactions between CXCR4 and LPA_1_ due to their C-terminal modifications, we examined the presence of CXCR4-LPA_1_ heteromers with a PLA. PLA signals were detected in HEK293A cells expressing Myc-CXCR4 and HA-LPA_1_ but not in cells expressing Myc-CXCR4 and HA-μOR or Myc-CXCR4 alone (Fig. 1H, I). To assess whether CXCR4-LPA_1_ heteromers are present in endogenous cells, we performed a PLA in the human triple-negative breast cancer cell line MDA-MB-231. We also utilized the CRISPR-Cas9 system to delete the *LPAR1* gene in MDA-MB-231 cells. As shown in Fig. 1J, both LPA_1_ knockout and control MDA-MB-231 cells exhibited strong single PLA signals for CXCR4 (Fig. 1K, left), suggesting that LPA_1_ expression does not affect cell surface expression of CXCR4. As expected, LPA_1_ knockout cells showed significantly reduced single PLA signals for LPA_1_ compared to those of control cells (Fig. 1K, middle). We observed strong double PLA signals for CXCR4-LPA_1_ heteromers in control MDA-MB-231 cells (Fig. 1J). However, double PLA signals for CXCR4-LPA_1_ heteromers were significantly reduced in LPA_1_ knockout cells (Fig. 1K, right). Taken together, these results demonstrate the presence of CXCR4-LPA_1_ heteromers in both recombinant HEK293A cells and an endogenous cell line MDA-MB-231.

### LPA_1_ inhibits CXCR4-mediated intracellular signaling

**Fig. 2:**
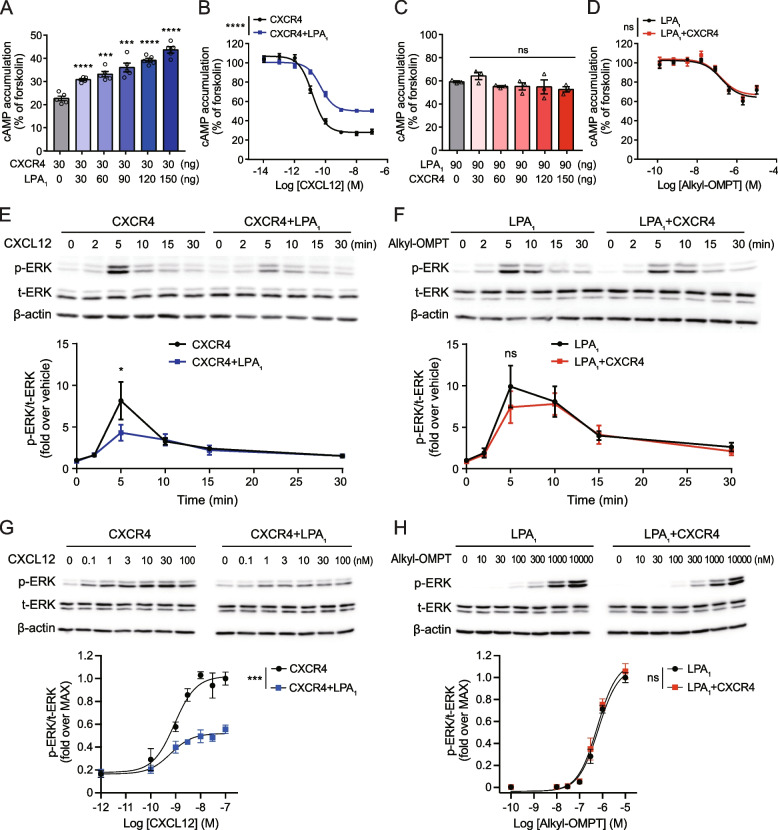
LPA_1_ reduces CXCL12-induced cAMP suppression and ERK phosphorylation. **A**-**D** Inhibition of the CXCL12-induced cAMP response by CXCR4-LPA_1_ heteromers. **A** HEK293A cells were transfected with Myc-CXCR4 alone or together with increasing amounts of Flag-LPA_1_, and the effect of CXCL12 (30 nM) on forskolin (3 μM)-induced cAMP production was measured using a GloSensor cAMP assay. **B** HEK293A cells were transfected with Myc-CXCR4 (30 ng) alone or together with Flag-LPA_1_ (120 ng) and CXCL12-induced inhibition of cAMP production was measured. **C** HEK293A cells were transfected with Flag-LPA_1_ alone or together with increasing amounts of Myc-CXCR4, and the effect of alkyl-OMPT (1 μM) on forskolin (3 μM)-induced cAMP production was measured. **D** HEK293A cells were transfected with Flag-LPA_1_ (90 ng) alone or together with Myc-CXCR4 (120 ng) and inhibition of cAMP production by alkyl-OMPT was measured. Data represent the mean ± SEM of *n* = 3 to 5 independent experiments performed in triplicate. Statistical significance was tested using unpaired two-tailed Student’s *t* test (**A**, **C**) or two-way ANOVA followed by Bonferroni’s multiple comparison test (**B**, **D**). ***P < 0.001; ****P < 0.0001; ns, not significant. **E**–**H** Inhibition of CXCL12-induced ERK phosphorylation by CXCR4-LPA_1_ heteromers. HEK293A cells were transfected with CXCR4 (20 ng), LPA_1_ (80 ng), or both and ERK phosphorylation induced by CXCL12 (10 nM) (**E**) or alkyl-OMPT (1 μM) (**F**) was examined. (**G**) CXCL12 dose–response for ERK phosphorylation was measured in HEK293A cells expressing CXCR4 alone or together with LPA_1_. **H** Alkyl-OMPT dose–response for ERK phosphorylation was measured in HEK293A cells expressing LPA_1_ alone or together with CXCR4. Data represent the mean ± SD of *n* = 3 to 5 independent experiments. Statistical significance was tested using two-way ANOVA followed by Bonferroni’s multiple comparison test. *P < 0.05; ***P < 0.001; ns, not significant

The activation of CXCR4 or LPA_1_ inhibits cAMP production through Gα_i/o_ activation. To investigate CXCR4-LPA_1_ heteromer-specific properties, the CXCR4-mediated cAMP response was analyzed in HEK293A cells transfected with CXCR4 plasmid alone or together with increasing amounts of LPA_1_ plasmid. We first checked the level of cell surface expression of each GPCR. Cell surface ELISA showed that approximately four times as many LPA_1_ plasmids as CXCR4 plasmids were required to achieve similar levels of cell surface expression for CXCR4 and LPA_1_ (Additional file 1: Fig. S2A, B). CXCL12 treatment reduced forskolin-induced cAMP production in cells expressing CXCR4 alone, and this CXCL12-induced inhibition was gradually reduced as the amount of transfected LPA_1_ plasmid was increased (Fig. 2A). The efficacy of CXCL12 was significantly reduced in the presence of LPA_1_, but the potency of CXCL12 was not affected (Fig. 2B). We next examined the LPA_1_-mediated cAMP response in HEK293A cells transfected with LPA_1_ plasmid alone or in combination with increasing amounts of CXCR4 plasmid. Stimulation of cells expressing LPA_1_ alone using alkyl-OMPT, an LPA_1_/LPA_3_-selective agonist, reduced forskolin-induced cAMP accumulation (Fig. 2C). Interestingly, the efficacy and potency of alkyl-OMPT were not different between cells expressing LPA_1_ alone and those expressing both LPA_1_ and CXCR4 (Fig. 2C, D).

To determine whether the inhibition of CXCR4 by LPA_1_ is due to decreased expression of CXCR4 by LPA_1_, the cell surface expressions of CXCR4 and LPA_1_ were analyzed by flow cytometry. Surface levels of CXCR4 and LPA_1_ increased as the quantities of transfected plasmids increased (Additional file 1: Fig. S2C, D). CXCR4 surface expression was not altered when HEK293A cells were cotransfected with increasing amounts of LPA_1_ plasmid (Additional file 1: Fig. S2E). Similarly, LPA_1_ surface expression was not affected by coexpression of CXCR4 (Additional file 1: Fig. S2F). These results suggest that the inhibition of CXCR4-mediated signaling by LPA_1_ is not due to decreased CXCR4 surface expression by LPA_1_.

To further characterize the properties of CXCR4-LPA_1_ heteromers, we investigated CXCL12- and alkyl-OMPT-induced ERK phosphorylation in HEK293A cells. CXCL12-induced rapid and transient ERK phosphorylation peaked 5 min after CXCL12 stimulation (Fig. 2E). Interestingly, ERK activation induced by CXCL12 was significantly reduced in cells expressing both CXCR4 and LPA_1_ compared to cells expressing CXCR4 alone. Alkyl-OMPT-induced ERK phosphorylation also peaked 5 min after stimulation and persisted longer than CXCL12-induced ERK phosphorylation (Fig. 2F). However, alkyl-OMPT-induced ERK phosphorylation in cells expressing LPA_1_ alone was not significantly different from that in cells expressing both LPA_1_ and CXCR4. Next, we analyzed the ligand dose–response for ERK phosphorylation to check whether LPA_1_ influences the E_Max_ or EC_50_ values of CXCL12. When we treated cells with CXCL12 for 5 min, the EC_50_ of CXCL12 was not significantly changed whether CXCR4 was expressed alone (0.87 ± 0.42 nM) or was coexpressed with LPA_1_ in cells (0.77 ± 0.52 nM) (Fig. 2G). Interestingly, the E_Max_ of CXCL12 was reduced approximately 50% in cells coexpressing both receptors compared to cells expressing CXCR4 alone. In contrast, the E_Max_ or EC_50_ values of alkyl-OMPT were not affected whether LPA_1_ was expressed alone or together with CXCR4 (Fig. 2H). Together, these results suggest that CXCR4 signaling is inhibited by heteromerization with LPA_1_ whereas LPA_1_ signaling is not affected by CXCR4.

### LPA_1_ interferes with CXCL12-induced G protein activation and β-arrestin recruitment to CXCR4

**Fig. 3:**
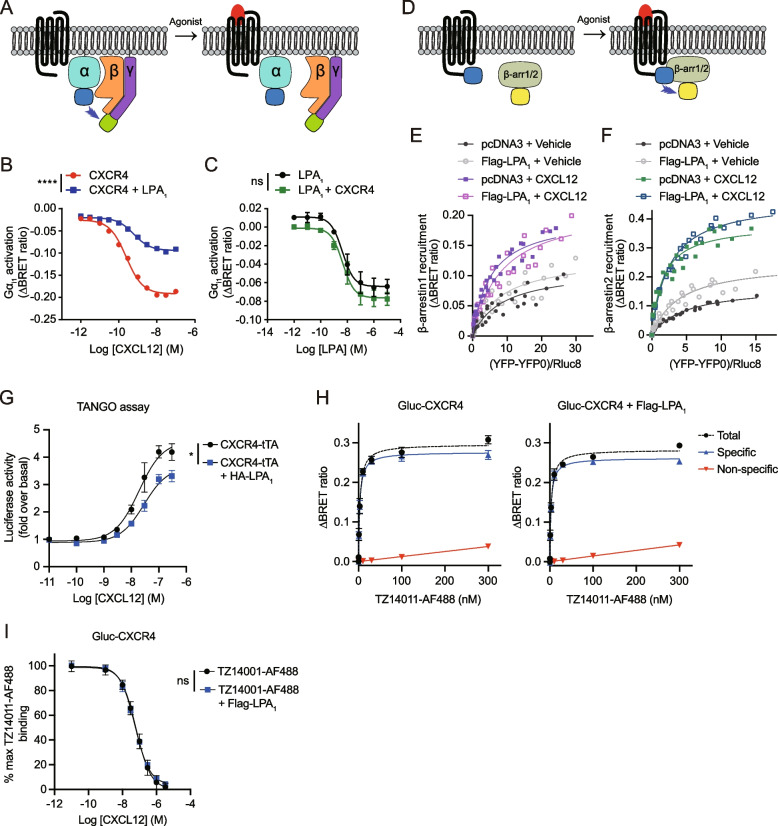
LPA_1_ inhibits CXCL12-induced Gα_i_ activation, β-arrestin recruitment, and ligand binding to CXCR4. **A** Schematic diagram of heterotrimeric G protein activation by BRET. **B** HEK293A cells were transfected with Myc-CXCR4 alone or together with Flag-LPA_1_ in the presence of Gα_i1_-Rluc8, Gβ_3_, and Gγ_9_-GFP2 (at a 1:1:1 DNA ratio), and CXCL12-induced Gα_i1_ activation was measured. **C** HEK293A cells were transfected with Flag-LPA_1_ alone or together with Myc-CXCR4 in the presence of Gα_i1_-Rluc8, Gβ_3_, and Gγ_9_-GFP2 (at a 1:1:1 DNA ratio), and LPA-induced Gα_i1_ activation was measured. Data represent the mean ± SEM of *n* = 3 to 4 independent experiments performed in triplicate. **D** Schematic diagram of β-arrestin recruitment to GPCR by BRET. **E**, **F** Ligand-induced β-arrestin1/2 BRET assay was performed in HEK293A cells. Vehicle or CXCL12 (50 nM)-induced ΔBRET increase between CXCR4-Rluc8 and mCitrine-β-arrestin1 (**E**) or mCitrine-β-arrestin2 (**F**) was measured. Data from *n* = 3 independent experiments (performed in triplicate) are presented as one site binding model. **G** PRESTO-Tango assay was performed for the recruitment of β-arrestin to CXCR4. Data represent the mean ± SEM of *n* = 4 independent experiments performed in duplicate. **H** Ligand binding assay was performed in HEK293A cells expressing Gluc-CXCR4 alone (Left panel) or together with Flag-LPA_1_ (Right panel). TZ14011-AF488 was used as a acceptor. IT1t (20 μM) was pretreated for the nonspecific BRET signal. The specific BRET signal was defined as the difference between the total BRET signal and the nonspecific BRET signal. **I** BRET-based competition binding experiments with CXCL12. After treatment with 10 nM TZ14011-AF488, unlabeled CXCL12 (0 to 3.3 μM) were applied for competition. Data represent the mean ± SEM of *n* = 3 to 4 independent experiments performed in duplicate. Statistical significance was tested by two-way ANOVA followed by Bonferroni’s multiple comparison test. *P < 0.05; ****P < 0.0001; ns, not significant

To analyze the inhibitory role of LPA_1_ toward CXCR4, we monitored G protein activation using a BRET-based biosensor [35]. Agonist-induced G protein activation was measured as a decrease in the BRET ratio between Gα_i_-Rluc8 and Gγ-GFP2 (Fig. 3A), reflecting dissociation of Gα from Gβγ or conformational rearrangement of the G protein complex [38, 39]. CXCL12 activated Gα_i1_, Gα_i2_, Gα_i3_, and Gα_oA_ in a dose-dependent manner, and their activation was significantly inhibited by coexpression of LPA_1_ (Fig. 3B, Additional file 1: Fig. S3A-C). In contrast, the activation of Gα_i1_, Gα_i2_, Gα_i3_, and Gα_oA_ induced by LPA or alkyl-OMPT was not inhibited by coexpression of CXCR4 (Fig. 3C, Additional file 1: Fig. S3D-J). To confirm that the effects of LPA_1_ depend on the heteromerization with CXCR4 rather than general sequestration of Gα_i/o_ proteins, we further examined whether the expression of μOR, which is also a Gα_i/o_-coupled receptor, affects CXCR4-mediated activation of G proteins. HEK293A cells expressing a constant amount of Myc-CXCR4 and increasing amounts of HA-μOR did not inhibit CXCL12-induced Gα_i3_ or Gα_oA_ activation (Additional file 1: Fig. S3K, L). In contrast, DAMGO, a μOR selective agonist, induced Gα_i3_ and Gα_oA_ activation in a manner dependent on the level of μOR expression (Additional file 1: Fig. S3M, N), demonstrating the functionality of μOR. We also examined the effect of μOR expression on CXCR4-mediated cAMP response. The expression of μOR did not affect CXCL12-induced inhibition of cAMP production (Additional file 1: Fig. S3O), while DAMGO-induced μOR activation inhibited forskolin-induced cAMP production (Additional file 1: Fig. S3P). These results suggest that LPA_1_ specifically inhibits CXCR4-mediated G protein activation, rather than non-selectively sequestering Gα_i/o_ proteins.

Next, we investigated agonist-induced β-arrestin recruitment (Fig. 3D). The BRET signal was measured in cells transfected with CXCR4-Rluc8 and mCitrine-β-arrestin in combination with or without LPA_1_. Ligand-induced change in BRET ratio could reflect an increased affinity of GPCR for β-arrestins or an alteration of GPCR-β-arrestin conformation that results in a longer or shorter distance between the luciferase and the fluorescent protein [40, 41]. Considering two possible outcomes, we performed a ligand-induced BRET saturation assay. We transfected HEK293A cells with a constant amount of CXCR4-Rluc8 plasmid and increasing amounts of mCitrine-β-arrestin1/2 plasmids. Even in the absence of CXCL12, a saturating basal BRET signal was observed for both β-arrestins (Fig. 3E, F). Expression of LPA_1_ led to decrease in BRET_50_ values compared to cells expressing pcDNA3 in a basal state, with β-arrestin2 exhibiting a more significant difference than β-arrestin1 (Table 2). These results indicate that the presence of LPA_1_ increases the propensity for the formations of CXCR4/β-arrestin complex without the necessity of CXCL12. Upon treatment with CXCL12, the ΔBRET_Max_ values for both β-arrestins increased compared to basal condition. Notably, this augmentation remained consistent regardless of the expression of LPA_1_. This increase is directly linked to changes in the distance and/or orientation between CXCR4-Rluc8 and mCitrine-β-arrestin. When LPA_1_ was not expressed, CXCL12 treatment led to a reduction in BRET_50_ values for both β-arrestins compared to those in the basal state. This implies that CXCR4 interacts more extensively with β-arrestins in the presence of CXCL12. When LPA_1_ was expressed, a slight increase in the CXCL12-induced BRET_50_ value was observed with β-arrestin1 compared to the basal state, whereas a decrease was observed with β-arrestin2. The CXCL12-induced BRET_50_ value for β-arrestin2 was much lower in cells where LPA_1_ was not expressed. Therefore, LPA_1_ appears to interfere with the interaction between CXCR4 and β-arrestins induced by CXCL12. To further substantiate the impact of LPA_1_ on β-arrestin recruitment to CXCR4, we employed a PRESTO-Tango assay [36]. HTLA cells were transfected with the CXCR4-Tango plasmid to assess whether coexpression with LPA_1_ would attenuate CXCL12-induced β-arrestin recruitment to CXCR4. We observed that cells coexpressing CXCR4-Tango and HA-LPA_1_ exhibited a slightly decreased potency (EC_50_ = 22.33 ± 6.65 nM) for CXCL12-induced β-arrestin recruitment to CXCR4, compared to cells expressing CXCR4-Tango alone (EC_50_ = 15.15 ± 5.27 nM) (Fig. 3G). In addition, the efficacy of CXCL12-induced β-arrestin recruitment to CXCR4 was reduced to approximately 80% in cells coexpressing CXCR4-Tango and HA-LPA_1_, as compared to cells only expressing CXCR4-Tango.

**Table 2:** BRET_Max_ and BRET_50_ values from the BRET β-arrestin saturation assay

		CXCR4-Rluc8 + pcDNA3		CXCR4-Rluc8 + Flag-LPA_1_	
		Vehicle	CXCL12	Vehicle	CXCL12
mCitrine- β-arrestin1	ΔBRET_Max_	0.111	0.198	0.131	0.217
	BRET_50_	8.639	5.273	8.071	8.469
mCitrine- β-arrestin2	ΔBRET_Max_	0.169	0.383	0.250	0.468
	BRET_50_	4.927	1.564	3.997	2.445

Heteromerization of GPCRs can alter the ligand binding affinity of their partner GPCRs via allosteric modulation [10, 11]. To investigate the effect of LPA_1_ on the binding of CXCL12 to CXCR4, we performed a BRET-based ligand binding assay utilizing Gluc [31] and the Alexa Fluor 488-conjugated CXCR4 antagonist TZ14011 (TZ14011-AF488). We expressed Gluc-tagged CXCR4 in HEK293A cells as a BRET donor and treated cells with TZ14011-AF488 as a BRET acceptor. To exclude the nonspecific signal caused by random collisions between donor and acceptor, the high-affinity CXCR4 antagonist IT1t was used as a competitor. In the absence of IT1t, increasing the concentration of TZ14011-AF488 led to a hyperbolic increase in the BRET ratio (Fig. 3H). In cells pretreated with 20 μM IT1t for 30 min, a linear increase in the BRET ratio was observed as the concentration of TZ14011-AF488 was increased. The specific BRET signal, which was defined as the difference between the total BRET signal and the one obtained in the presence of IT1t, was saturated at nanomolar concentration, demonstrating the high affinity and specific interaction of TZ14011-AF488 with Gluc-CXCR4. When Flag-LPA_1_ was coexpressed with Gluc-CXCR4, the specific BRET ratio between Gluc-CXCR4 and TZ14011-AF488 was similar to that in cells expressing Gluc-CXCR4 alone (ΔBRET_Max_ = 0.277 ± 0.008 in cells expressing Gluc-CXCR4 alone; ΔBRET_Max_ = 0.262 ± 0.01 in cells coexpressing Gluc-CXCR4 and Flag-LPA_1_) (Fig. 3H). The equilibrium dissociation constant (*K*_D_) values between Gluc-CXCR4 and TZ14011-AF488 were also comparable regardless of Flag-LPA_1_ coexpression (*K*_D_ = 2.94 ± 0.46 nM in cells expressing Gluc-CXCR4 alone; *K*_D_ = 2.77 ± 0.56 nM in cells coexpressing Gluc-CXCR4 and Flag-LPA_1_). Based on the ligand binding saturation experiments described above, we conducted TZ14011-AF488 competition binding experiments, wherein cells were treated with increasing concentrations of unlabeled CXCL12 to measure the dissociation of TZ14011-AF488 from Gluc-CXCR4. As shown in Fig. 3I, similar concentrations of CXCL12 were required to dissociate TZ14011-AF488 from Gluc-CXCR4 irrespective of Flag-LPA_1_ coexpression (IC_50_ = 62.11 ± 5.42 nM in cells expressing Gluc-CXCR4 alone; IC_50_ = 54.52 ± 7.76 nM in cells coexpressing Gluc-CXCR4 and Flag-LPA_1_). Taken together, these results suggest that while heteromerization between CXCR4 and LPA_1_ does not affect the affinity of CXCL12 for CXCR4, coexpression of LPA_1_ with CXCR4 is sufficient to affect CXCR4-mediated Gα_i/o_ and β-arrestin-dependent signaling pathways.

### LPA inhibits CXCL12-induced Gα_i_ recruitment to CXCR4

**Fig. 4:**
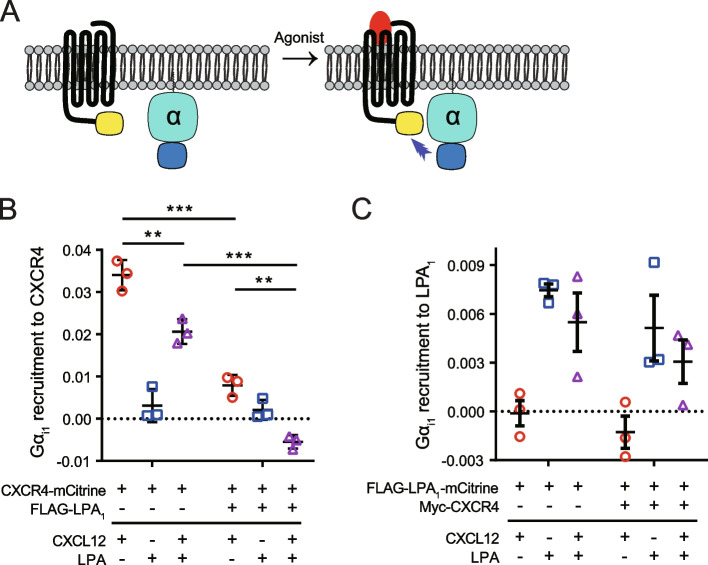
LPA reduces CXCR4-mediated Gα_i_ activation. **A** Schematic diagram of measuring Gα_i_ recruitment to GPCR by BRET. **B** HEK293A cells were cotransfected with Gα_i1_-Rluc8, Gβ_1_, Gγ_1_, and CXCR4-mCitrine alone or together with Flag-LPA_1_. BRET between CXCR4-mCitrine and Gα_i1_-Rluc8 was measured after stimulation with CXCL12 (10 nM) or LPA (1 μM). **C** HEK293A cells were cotransfected with Gα_i1_-Rluc8, Gβ_1_, Gγ_1_, and Flag-LPA_1_-mCitrine alone or together with Myc-CXCR4. BRET between Flag-LPA_1_-mCitrine and Gα_i1_-Rluc8 was measured after stimulation with CXCL12 (10 nM) or LPA (1 μM). Data represent the mean ± SEM of *n* = 3 independent experiments performed in triplicate. Statistical significance was tested using unpaired two-tailed Student’s *t* test. **P < 0.01; ***P < 0.001

Because both CXCR4 and LPA_1_ are Gα_i/o_-coupled receptors, CXCR4-mediated signaling cannot be distinguished from LPA_1_ signaling based on cAMP measurement assays or G protein activation assays upon costimulation of both receptors. To examine the effect of LPA on CXCL12-induced Gα_i1_ signaling, Gα_i1_ recruitment to CXCR4 was analyzed by BRET in HEK293A cells in the presence or absence of LPA_1_ (Fig. 4A). CXCL12, but not LPA, induced an increase in BRET signal between Gα_i1_-Rluc8 and CXCR4-mCitrine, implying that Gα_i1_ recruitment to CXCR4 is specifically induced by CXCL12 (Fig. 4B). Notably, costimulation of cells with both CXCL12 and LPA reduced CXCL12-induced Gα_i1_ recruitment to CXCR4. The inhibitory effect of LPA on CXCL12-stimulated Gα_i1_ signaling in HEK293A cells transfected with CXCR4 alone seems to reflect the presence of endogenous LPA_1_. Consistent with our findings described above, CXCL12-induced Gα_i1_ recruitment to CXCR4 was considerably inhibited in cells coexpressing CXCR4 and LPA_1_. Costimulation with LPA further decreased CXCL12-induced Gα_i1_ recruitment to CXCR4 in the presence of LPA_1_, suggesting an allosteric modulation of CXCR4-Gα_i1_ signaling by LPA. In contrast, LPA-induced Gα_i1_ recruitment to LPA_1_ was not significantly affected by CXCL12 or CXCR4 (Fig. 4C). These results suggest that LPA unidirectionally inhibits CXCR4 function through allosteric regulation of CXCR4 in cells expressing both CXCR4 and LPA_1_.

### Loss of *LPAR1* in MDA-MB-231 cells increases CXCR4-mediated calcium signaling and cell migration

**Fig. 5:**
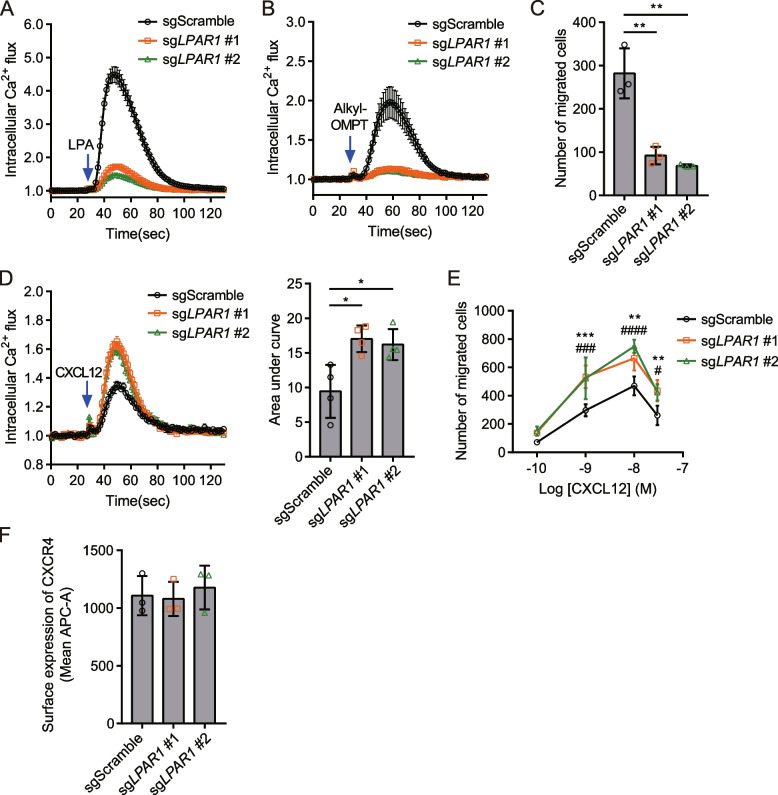
Enhanced CXCR4 responses in MDA-MB-231 cells targeted with sg*LPAR1* using the CRISPR-Cas9 gene editing system. **A**, **B** MDA-MB-231 cells were transduced with sgScramble, sg*LPAR1* #1, or sg*LPAR1* #2, and transduced cells were selected with puromycin for two weeks. To evaluate LPA_1_ deficiency, LPA (1 μM, A) or alkyl-OMPT (1 μM, B)-induced calcium flux was measured in cells targeted with sgScramble or sg*LPAR1*. **C** LPA_1_-mediated migration was measured using a transwell migration assay with alkyl-OMPT (1 μM) in cells treated with sgScramble or sg*LPAR1*. **D** Intracellular calcium flux induced by CXCL12 (10 nM) was measured in cells targeted with sgScramble or sg*LPAR1* (Left). The area under curve was measured for CXCL12-induced intracellular calcium levels (Right). **E** CXCL12-induced migration was evaluated in in cells targeted with sgScramble or sg*LPAR1*. **F** Cell surface expression of CXCR4 was measured by flow cytometry with anti-CXCR4 (4G10) primary antibody and anti-mouse antibody conjugated with APC. **C**, **D** Statistical significance was tested using unpaired two-tailed Student’s *t* test. *P < 0.05; **P < 0.01. **C**, **E** Migrated cells were counted from randomly selected images of 5 fields. Data represent the mean ± SD of *n* = 3 to 4 independent experiments performed in triplicate. **E** Statistical significance was tested using two-way ANOVA followed by Bonferroni’s multiple comparison test comparing “sgScramble” to “sg*LPAR1* #1” (**P < 0.01; ***P < 0.001) and “sgScramble” to “sg*LPAR1* #2” (#*P* < 0.05; ###*P* < 0.001; ####*P* < 0.0001)

To investigate the role of the CXCR4-LPA_1_ heteromer in cancer cells, we first measured the expression of CXCR4 and LPA receptor subtypes in MDA-MB-231 cells using real-time quantitative PCR (RT-qPCR). Significant levels of *CXCR4* and *LPAR1* mRNA were detected in MDA-MB-231 cells, whereas mRNA for other LPA receptors was barely detectable (Additional file 1: Fig. S4A). We next examined whether LPA_1_ is responsible for CXCR4 inhibition in MDA-MB-231 cells by deleting *LPAR1* with the CRISPR/Cas9 system. We generated *LPAR1* knockout MDA-MB-231 pool cells by transducing lentiviruses encoding *LPAR1*-targeting single guide RNAs (sg*LPAR1* #1 and #2). We validated *LPAR1* knockout at the gene level using restriction enzymes that target the PAM sites for sg*LPAR1* #1 and #2 (Additional file 1: Fig. S4B, C), as well as Sanger sequencing analysis (Additional file 1: Fig. S4D). In *LPAR1* knockout MDA-MB-231 cells, intracellular calcium flux induced by LPA or alkyl-OMPT was significantly reduced (Fig. 5A, B). Alkyl-OMPT-induced cell migration was also significantly reduced in MDA-MB-231 cells targeted with sg*LPAR1* compared to cells targeted with the sgScramble control (Fig. 5C). These observations suggest that functional LPA_1_ has been efficiently depleted in sg*LPAR1*-targeted MDA-MB-231 cells. Remarkably, CXCL12-induced calcium flux was significantly increased in LPA_1_-deficient MDA-MB-231 cells compared to control cells (Fig. 5D). MDA-MB-231 cells targeted with sg*LPAR1* also exhibited significantly increased migration toward CXCL12 (Fig. 5E). There was no difference in CXCR4 expression between cells transduced with sgScramble or sg*LPAR1* (Fig. 5F*,* Additional file 1: Fig. S4E), implying that increased calcium flux and migration upon CXCL12 treatment in *LPAR1* knockout cells are not due to increased surface expression of CXCR4. Taken together, these results demonstrate that LPA_1_ has an inhibitory role on CXCR4 function in MDA-MB-231 cells, which endogenously express both receptors.

### LPA_1_ activation interferes with CXCL12-induced migration in cancer cells

**Fig. 6:**
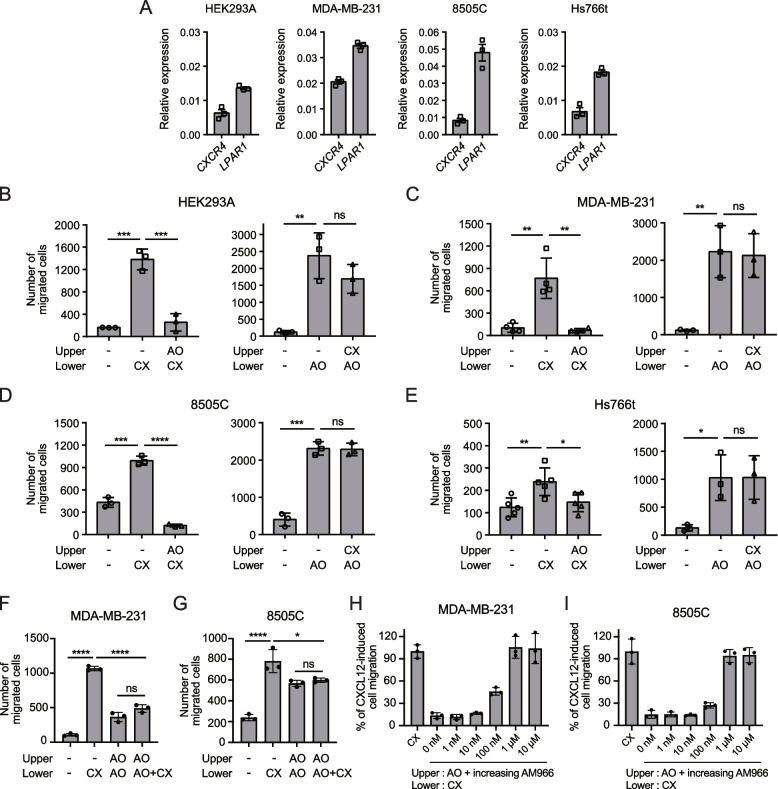
Effect of LPA_1_ activation on CXCR4-mediated migration in cell lines endogenously expressing both receptors. **A** Expression of *CXCR4* and *LPAR1* in HEK293A, MDA-MB-231, 8505C, and Hs766t cells was measured using RT-qPCR. The relative expression level of each GPCR was normalized to that of GAPDH. Data represent the mean ± SEM of *n* = 3 independent experiments. **B**-**E** The effect of LPA_1_ stimulation by alkyl-OMPT on CXCL12-induced cell migration was assessed using a transwell migration assay with alkyl-OMPT (1 μM) in the upper chamber and CXCL12 (10 nM) in the lower chamber (Left panels). The effect of CXCR4 stimulation by CXCL12 on alkyl-OMPT-induced cell migration was assessed using a transwell migration assay with CXCL12 (10 nM) in the upper chamber and alkyl-OMPT (1 μM) in the lower chamber (Right panels). Migrated cells were counted from randomly selected images of 10 fields. Data represent the mean ± SD of *n* = 3 to 5 independent experiments. **F**, **G** The effect of alkyl-OMPT (1 μM) in both the upper and lower chambers on CXCL12 (10 nM)-induced cell migration was assessed in MDA-MB-231 (**F**) and 8505C cells (**G**). Data represent the mean ± SD of *n* = 3 independent experiments. Statistical significance was tested using an unpaired two-tailed Student’s *t* test. *P < 0.05; **P < 0.01; ***P < 0.001; ****P < 0.0001; ns, not significant. **H**, **I** CXCL12 (10 nM)-induced cell migration was assessed in the presence of alkyl-OMPT (1 μM) with increasing concentrations of AM966 in the upper chamber. Data represent the mean ± SD of *n* = 3 independent experiments. AO: alkyl-OMPT; CX: CXCL12

CXCR4 is widely overexpressed in various cancer cells, including MDA-MB-231 breast cancer cells [42], 8505C thyroid cancer cells [43], Hs766t pancreatic cancer cells [44], Jurkat T cells, and U937 and THP-1 monocytic leukemia cells [45, 46]. To investigate whether ligand-activated LPA_1_ inhibits CXCR4 in these cells, we first checked the mRNA expression of CXCR4 and LPA receptor subtypes using RT-qPCR. *CXCR4* was expressed in all cells tested except for MCF7, while *LPAR1* expression was observed in HEK293A, MDA-MB-231, 8505C, and Hs766t cells (Fig. 6A*,* Additional file 1: Fig. S4A, S5). We also found that LPA_1_ is a major LPA receptor expressed in HEK293A, MDA-MB-231, 8505C, and Hs766t cells. In contrast, U937, THP-1, and Jurkat cells expressed *CXCR4* along with negligible levels of *LPAR1-6*.

To examine whether LPA_1_ activation with alkyl-OMPT or LPA alters cell migration toward CXCL12, we performed a transwell migration assay. Consistent with CXCR4 expression, CXCL12-induced migration was observed in all cells except for MCF7 cells (Fig. 6B-E, Additional file 1: Fig. S6). Similarly, alkyl-OMPT-induced migration was observed in HEK293A, MDA-MB-231, 8505C, and Hs766t cells that express LPA_1_, but not in MCF7, U937, THP-1, and Jurkat cells that do not express LPA_1_. Interestingly, the addition of alkyl-OMPT to the upper chamber inhibited the migration of HEK293A, MDA-MB-231, 8505C, and Hs766t cells, which endogenously express both CXCR4 and LPA_1_, toward CXCL12 in the lower chamber (Fig. 6B-E). In contrast, the addition of CXCL12 to the upper chamber did not reduce cell migration toward alkyl-OMPT in the lower chamber in these cells. Similar to alkyl-OMPT, LPA in the upper chamber also reduced the migration of MDA-MB-231 and 8505C cells toward CXCL12 in the lower chamber, while CXCL12 did not affect LPA-induced cell migration (Additional file 1: Fig. S7A, B). These results indicate that the inhibitory effect of LPA_1_ on CXCR4 is unidirectional. Alkyl-OMPT did not affect CXCL12-induced migration in U937, THP-1, and Jurkat cells that do not express LPA_1_ (Additional file 1: Fig. S6), suggesting that the effect of alkyl-OMPT on CXCL12-induced migration is mediated by LPA_1_.

To rule out the possibility of cell sequestration in the upper chamber, we examined LPA_1_ agonists on CXCL12-induced cell migration in the presence of alkyl-OMPT or LPA in both chambers. When the same concentration of alkyl-OMPT or LPA was placed in both chambers, it increased the migration of MDA-MB-231 and 8505C cells toward the lower chamber (Fig. 6F, G, Additional file 1: Fig. S7C, D). This could possibly be due to the enhancement of cell movement in the absence of a chemoattractant gradient [47]. Remarkably, even with a CXCL12 gradient directed toward the lower chamber, the LPA_1_ agonist present in both chambers completely suppressed the CXCL12-induced cell migration toward the lower chamber. These results unequivocally illustrate that the inhibition of CXCR4-mediated migration by the LPA_1_ agonist is not a consequence of cell sequestration in the upper chamber, but rather a direct inhibitory effect on CXCR4 by LPA_1_.

To confirm our finding that LPA_1_ activation inhibits CXCR4-mediated cell migration, we also performed a transwell migration assay in the presence of both alkyl-OMPT and AM966 in the upper chamber. The addition of the LPA_1_-selective antagonist AM966 to the upper chamber fully reversed the inhibitory effect of alkyl-OMPT on CXCR4-mediated migration of MDA-MB-231 and 8505C cells (Fig. 6H, I). Taken together, these results suggest that the LPA_1_ activation inhibits CXCL12-induced migration in cancer cells that endogenously express both CXCR4 and LPA_1_.

### LPA_1_ antagonists restore LPA_1_-mediated suppression of CXCR4 function

**Fig. 7:**
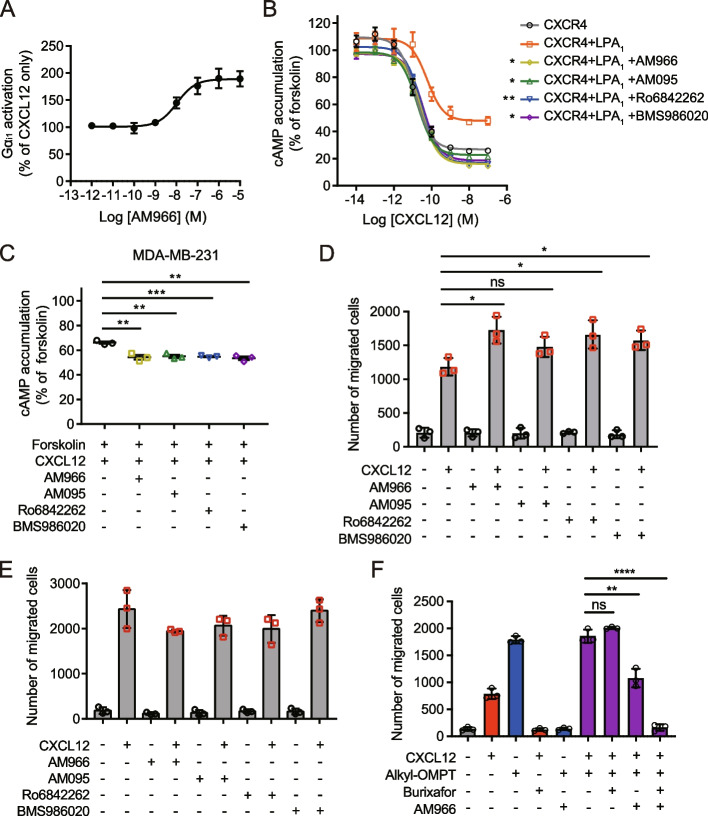
LPA_1_ antagonists increase CXCR4 signaling and function. **A** HEK293A cells were transfected with CXCR4 and LPA_1_ and pretreated with AM966 at the indicated concentrations for 30 min. CXCL12 (10 nM)-induced Gα_i1_ activation was measured using BRET between Gα_i1_-Rluc8 and Gγ_9_-GFP2. **B** HEK293A cells were transfected with CXCR4 alone or together with LPA_1_ and pretreated with LPA_1_ antagonists AM966, AM095, Ro6842262, or BMS986020 at 10 μM for 30 min. Cells were treated with forskolin (3 μM) and CXCL12 (30 nM), and the effects of LPA_1_ antagonists on CXCR4-mediated cAMP responses were measured using the GloSensor cAMP assay. **C** MDA-MB-231 cells were pretreated with LPA_1_ antagonists at 10 μM for 30 min, and CXCL12 (30 nM)-induced inhibition of forskolin (3 μM)-induced cAMP accumulation was measured using the GloSensor cAMP assay. **D**, **E** The parental MDA-MB-231 (**D**) or *LPAR1* knockout MDA-MB-231 cells (**E**) were pretreated with LPA_1_ antagonists at 10 μM for 30 min, and CXCL12 (10 nM)-induced migration was examined using a transwell migration assay. **F** MDA-MB-231 cells were pretreated with burixafor (1 μM) and/or AM966 (10 μM) and assessed for cell migration toward CXCL12 (10 nM) and/or alkyl-OMPT (1 μM) using a transwell migration assay. Migrated cells were counted from randomly selected images of 10 fields. **A**-**C** Data represent the mean ± SEM of *n* = 3 to 4 independent experiments performed in triplicate. **D**-**F** Data represent the mean ± SD of *n* = 3 independent experiments performed in triplicate. **B** Statistical significance was tested using two-way ANOVA followed by Bonferroni’s multiple comparison test (DMSO vs. LPA_1_ antagonists). *P < 0.05; **P < 0.01. **C**, **D**, and **F** Statistical significance was tested using unpaired two-tailed Student’s *t* test. *P < 0.05; **P < 0.01; *** P < 0.001; ****P < 0.0001; ns, not significant

Given that LPA_1_ activation inhibits CXCR4 function, we next examined whether LPA_1_ antagonists can restore LPA_1_-mediated suppression of CXCR4 function. Pretreatment with the LPA_1_-selective antagonists AM966, AM095, Ro6842262, and BMS986020 increased CXCL12-induced Gα_i1_ activation in a dose-dependent manner in HEK293A cells transfected with CXCR4 and LPA_1_ (Fig. 7A*,* Additional file 1: Fig. S8). LPA_1_ antagonists also increased the CXCL12-induced inhibition of cAMP production in HEK293A cells coexpressing CXCR4 and LPA_1_ (Fig. 7B). Consistent with these results, pretreatment with LPA_1_ antagonists enhanced the CXCL12-induced inhibition of cAMP production in MDA-MB-231 cells (Fig. 7C). In addition, LPA_1_ antagonists increased CXCL12-induced migration in the parental MDA-MB-231 cells (Fig. 7D) but not in LPA_1_-deficient MDA-MB-231 cells (Fig. 7E), suggesting that increased cell migration by LPA_1_ antagonists is mediated by LPA_1_. These results also raise the possibility that LPA_1_ antagonists can enhance CXCR4-mediated migration of cells coexpressing both receptors, thus exacerbating cancer cell migration and metastasis. Finally, we examined the effect of CXCR4 and LPA_1_ antagonists on cell migration induced by costimulation of CXCR4 and LPA_1_. The CXCR4-selective antagonist burixafor [48] and the LPA_1_-selective antagonist AM966 each completely prevented cell migration induced by CXCL12 and alkyl-OMPT, respectively (Fig. 7F). MDA-MB-231 cell migration induced by cotreatment with CXCL12 and alkyl-OMPT was similar to that induced by alkyl-OMPT alone, reflecting the inhibition of CXCL12-induced migration by LPA_1_ activation. AM966 completely inhibited cell migration induced by alkyl-OMPT alone, whereas it did not completely inhibit cell migration induced by cotreatment with alkyl-OMPT and CXCL12. Although burixafor alone did not affect cell migration induced by cotreatment with alkyl-OMPT and CXCL12, it completely inhibited migration induced by alkyl-OMPT and CXCL12 cotreatment in the presence of AM966. Taken together, these results suggest that, when CXCR4 and LPA_1_ are activated simultaneously, net cell migration may not differ from migration regulated by individual receptors, but both antagonists are required to fully inhibit cell migration.

### *CXCR4* expression is not correlated with poor survival in cancers with high *LPAR1* expression

CXCR4 is known to be overexpressed in more than 23 human cancers, and a negative correlation between CXCR4 expression and OS has been shown in non-small cell lung cancer, colorectal cancer, renal cell carcinoma, and acute myelogenous leukemia [18, 49–52]. CXCR4 has also been identified as a significant unfavorable prognostic marker in renal and stomach cancers among the 17 major human cancer types based on the TCGA data [53]. However, it is notable that high CXCR4 expression was found to be significantly favorable in ovarian cancer in that study. So far, we showed that LPA and alkyl-OMPT inhibit CXCR4-mediated migration of breast, thyroid, and pancreatic cell lines endogenously expressing CXCR4 and LPA_1_ (Fig. 6, Additional file 1: Fig. S7). Given these results, we analyzed the relationship between *CXCR4* and *LPAR1* expression and OS using the TCGA datasets. In breast cancer, high *CXCR4* expression was associated with a favorable survival compared to low *CXCR4* expression (Additional file 1: Fig. S9A, left). No difference in OS was observed between the *CXCR4*-high and *CXCR4*-low groups in thyroid and pancreatic cancers (Additional file 1: Fig. S9B, C, left). Patients expressing high levels of *CXCR4* in these cancers also had high levels of *LPAR1* (Additional file 1: Fig. S9A-C, right). Presumably, CXCR4 inhibition by LPA_1_ may play a role in the lack of correlation between *CXCR4* expression and OS in these cancers. In contrast, *CXCR4* expression was negatively associated with OS in acute myelogenous leukemia (Additional file 1: Fig. S9D, left). In this cancer, there was no difference in *LPAR1* expression between the *CXCR4*-high and *CXCR4*-low groups (Additional file 1: Fig. S9D, right). Taken together, these results suggest that CXCR4 function may be inhibited by LPA_1_ in cancer tissues as well as in cancer cell lines.

## Discussion

Growing evidence indicates that class A GPCRs can form homomers and heteromers, which exhibit distinct functional and pharmacological properties not observed in individual receptors [54]. The possibility of a physical interaction between CXCR4 and LPA_1_ has been reported from a large-scale protein–protein interaction study using affinity purification-mass spectrometry [55]. In a preliminary study, we also identified LPA_1_ as a CXCR4 interactor using an adenovirus-based BiFC screen [30, 34]. However, the functional roles of CXCR4-LPA_1_ heteromers were poorly understood. In this study, we demonstrated that LPA_1_ unidirectionally inhibits CXCR4 not only in the heterologous expression system but also in various cancer cells endogenously expressing both receptors. We found that LPA_1_ interferes with CXCR4-mediated Gα_i/o_ activation, cAMP signaling, β-arrestin recruitment, and ERK activation. LPA completely inhibited CXCL12-induced Gα_i1_ recruitment to CXCR4, whereas CXCL12 did not affect LPA-induced Gα_i1_ recruitment to LPA_1_. Deletion of *LPAR1* in MDA-MB-231 cells increased CXCL12-induced calcium flux and cell migration, indicating that LPA_1_ can inhibit CXCR4 in cancer cells expressing both receptors. The inhibition of CXCR4-mediated migration in breast, thyroid, and pancreatic cancer cells by LPA implies that LPA can exert a negative regulatory effect on CXCR4 function in cancer and inflammatory conditions where LPA is abundtant. This finding highlights the potential therapeutic relevance of targeting the CXCR4-LPA_1_ axis in such disease contexts. Our research also demonstrated that LPA_1_ antagonists enhance CXCL12-induced signaling and cell migration in a LPA_1_-dependent manner. This finding raises concerns regarding the use of LPA_1_ antagonists alone in conditions where both CXCR4 and LPA_1_ are coexpressed and contribute to disease progression. Instead our results strongly suggest that a combination of CXCR4 and LPA_1_ antagonists could provide a more effective inhibition of functions mediated by both receptors, offering a prospective therapeutic approach for diseases that involve the CXCR4-LPA_1_ axis.

In this study, we validated the formation of CXCR4-LPA_1_ heteromers using BiFC, BRET, and PLAs in both recombinant system and endogenous cancer cells. In fact, CXCR4 is known to form not only homodimers [56] but also heteromers with other chemokine receptor family members or with other families of GPCRs. Heteromerization among CXCR4, CCR2, and CCR5 results in bidirectional negative ligand binding cooperativity and functional cross-inhibition in terms of calcium mobilization and chemotaxis of leukocytes [57, 58]. Upon stimulation with CXCL12, CXCR4 inhibits CXCR1-, CXCR3-, CXCR5-, CXCR6-, and CCR2-mediated migration of human immune cells when coexpressed on the cell surface [59], suggesting that CXCL12/CXCR4 signaling is dominant over other chemokine signaling. Agonist stimulation induces heteromerization between CXCR4 and CB2 in human breast and prostate cancer cells, resulting in decreased calcium signaling and cell migration [60]. Simultaneous activation of Gα_i/o_-coupled CXCR4-δ-opioid receptor (δOR) heteromers results in cross-inhibition of both receptors without affecting ligand binding or receptor expression [61]. Pretreatment with a δOR antagonist restores CXCR4 function in μOR-deficient mouse brain homogenates and brain slices [62]. This negative antagonism induced by simultaneous activation of both receptors in several GPCR heteromers has been explained by steric hindrance between the interacting transmembrane domains, which does not allow full opening of transmembrane domains 5 and 6 to efficiently accommodate G proteins [63]. Heteromerization of CXCR4 with ADRA1A/ADRA1B has been found on the surface of vascular smooth muscle cells, and activation of CXCR4 increases the potency of α1-adrenergic agonists on the blood pressure response in rats [64]. As shown in these reports, CXCR4 heteromerization alters the pharmacological properties of CXCR4. Our results of BRET saturation experiments suggest that CXCR4 homomers are formed with the highest efficiency, while CXCR4-LPA_1_ heteromers are formed more efficiently than LPA_1_ homomers in cells (Table 1). Therefore, it is likely that the inhibition of CXCR4 signaling by CXCR4-LPA_1_ heteromers is limited to a certain extent. Although LPA_1_ expression did not modify the ligand binding affinity of CXCR4, we observed an enhancement in the preform of CXCR4/β-arrestins under basal conditions. These results exclude the possibility that LPA_1_-mediated suppression of CXCR4 functions could be a consequence of inhibited CXCL12 binding to CXCR4. Considering the notion that the G proteins and β-arrestin engage in competitive binding with GPCR, the observed increase in the preform of CXCR4/β-arrestin in the basal state seems to have influenced Gα_i/o_ signaling of CXCR4 induced by CXCL12. Given that CXCR4 is an important therapeutic target for the treatment of cancer and immune-related diseases, further identification of CXCR4 heteromers and comprehensive understanding of their physiological relevance will provide valuable insights for the advancement of future therapeutics.

Suppression of CXCR4-mediated migration by LPA_1_ suggests a signaling hierarchy in which LPA_1_-mediated signaling can override signaling pathways downstream of CXCR4. Although CXCR4 is highly overexpressed in a variety of cancers, CXCR4 expression does not correlate with poor OS in many cancer types. We found that patients expressing high levels of CXCR4 have high levels of LPA_1_ in breast, pancreatic, and thyroid cancers, suggesting that the lack of correlation between CXCR4 expression and patient survival may be due to CXCR4 inhibition by the LPA_1_/LPA axis. LPA_1_ is known to be expressed in NK cells and is involved in NK-cell migration and IFN-γ secretion [65]. LPA_1_ expression is positively correlated with the infiltration of immune cells, including CD4^+^ and CD8^+^ T cells, NK cells, and dendritic cells, leading to improved OS in prostate cancer [66]. Given that CXCR4 antagonists increase CD8^+^ T cell infiltration into tumor tissues and the expression of CXCR4 in these cells [67], it is plausible that the recruitment of pro-inflammatory immune cells induced by LPA_1_ is partially due to LPA_1_’s inhibitory effect on CXCR4 in these cells.

LPA_1_ has been implicated in cancer invasion, lung fibrosis, autoimmune disorders, hydrocephalus, and neuropathic pain, and LPA_1_ antagonists have been under development for treating autoimmune diseases and cancers [68, 69]. AM966 and VPC12249 have shown efficacy in murine idiopathic pulmonary fibrosis [70, 71], and BMS986020 has finished phase II clinical trials for idiopathic pulmonary fibrosis [72]. The LPA_1_/LPA_3_ dual antagonist SAR100842 has completed phase II clinical trials for systemic sclerosis [73], and the LPA_1_/LPA_3_ inhibitors Ki16425, Ki16198, and Debio 0719 have been tested in mouse cancer models [74–76]. Despite numerous efforts, no LPA_1_-targeting drugs have been approved by the FDA. Our data raise the possibility that while LPA_1_ antagonists attenuate LPA-induced responses, they may amplify CXCR4-mediated responses in cells or tissues where both GPCRs are coexpressed. To avoid the stimulatory effects of LPA_1_ antagonists on CXCR4 function, it will be advantageous to use LPA_1_ antagonists in combination with CXCR4 inhibitors.

## Conclusion

Our study elucidates the formation of heteromers betewwn CXCR4 and LPA_1_, leading to the inhibition of CXCR4-mediated signaling and cell migration through the expression and activation of LPA_1_. These findings contribute to an enhanced understanding of the molecular mechanisms modulating CXCR4 function in the context of cancer and inflammatory diseases. Furthermore, our results propose a promising approach of using both CXCR4 and LPA_1_ antagonists in diseases that involve the CXCR4-LPA_1_ axis. This combination could potentially provide a therapeutic strategy with broader efficacy in targeting these pathways.

### Supplementary Information

**Additional file 1: Additional file 1: Fig. S1.** BRET saturation assay performed in HEK293A cells. **Fig. S2.** Cell surface expression of CXCR4 and LPA_1_. **Fig. S3.** Evaluation of heterotrimeric G protein activation. **Fig. S4.** Expression of GPCRs in MDA-MB-231 cells and validation of *LPAR1* knockout. **Fig. S5.** Expression of CXCR4 and LPA receptors in various human cell lines. **Fig. S6.** CXCR4-mediated migration in cell lines that do not express LPA_1_. **Fig. S7.** The effect of LPA stimulation on CXCR4-mediated cell migration. **Fig. S8.** The effect of LPA_1_ antagonists on CXCL12-induced G protein activity. **Fig. S9.** Overall survival and expression analysis of *CXCR4 *and *LPAR1* in the TCGA datasets.

## Data Availability

All data generated or analyzed during this study are included in this published article and its supplementary information files.

## Abbreviations

AdBiFC: Adenovirus-based bimolecular fluorescence complementation
BiFC: Bimolecular fluorescence complementation
BRET: Bioluminescence resonance energy transfer
CB2: Cannabinoid receptor 2
CXCR4: CXC chemokine receptor 4
ERK1/2: Extracellular signal-regulated kinase 1/2
FDA: Food and Drug Administration
Gluc: *Gaussia* Luciferase
GPCRs: G protein-coupled receptors
LPA: Lysophosphatidic acid
LPA_1_: Lysophosphatidic acid receptor 1
MAPK: Mitogen-activated protein kinase
OS: Overall survival
PKB/Akt: Protein kinase B
PLA: Proximity ligation assay
S1P_1_: Sphingosine-1-phosphate receptor 1
SLIC: Sequence- and ligation-independent cloning
TCGA: The Cancer Genome Atlas
δOR: δ-Opioid receptor
μOR: μ-Opioid receptor
