# Supplementary material for: LPA1-mediated inhibition of CXCR4 attenuates CXCL12-induced signaling and cell migration
Source: Cell Commun Signal. 2023 Sep 25;21:257. doi: 10.1186/s12964-023-01261-7 (PMC10518940; doi:10.1186/s12964-023-01261-7)
Supplement: Supplementary file 2 — Additional file 1: Additional file 1: Fig. S1. BRET saturation assay performed in HEK293A cells. Fig. S2. Cell surface expression of CXCR4 and LPA1. Fig. S3. Evaluation of heterotrimeric G protein activation. Fig. S4. Expression of GPCRs in MDA-MB-231 cells and validation of LPAR1 knockout. Fig. S5. Expression of CXCR4 and LPA receptors in various human cell lines. Fig. S6. CXCR4-mediated migration in cell lines that do not express LPA1. Fig. S7. The effect of LPA stimulation on CXCR4-mediated cell migration. Fig. S8. The effect of LPA1 antagonists on CXCL12-induced G protein activity. Fig. S9. Overall survival and expression analysis of CXCR4 and LPAR1 in the TCGA datasets. [file 12964_2023_1261_MOESM1_ESM.docx]

**Supplementary Information for**

LPA_1_-mediated inhibition of CXCR4 attenuates CXCL12-induced signaling and cell migration

Jong Min Hong^1^, Jin-Woo Lee^1^, Dong-Seung Seen^2^, Jae-Yeon Jeong^2^*, Won-Ki Huh^1,3^*

^1^School of Biological Sciences, Seoul National University, Seoul 08826, Republic of Korea

^2^GPCR Therapeutics Inc., Gwanak-gu, Seoul 08790, Republic of Korea

^3^Institute of Microbiology, Seoul National University, Seoul 08826, Republic of Korea

*Corresponding authors: Jae-Yeon Jeong ([jeongjy@gpcr.co.kr](mailto:jeongjy@gpcr.co.kr)) and Won-Ki Huh ([wkh@snu.ac.kr](mailto:wkh@snu.ac.kr))


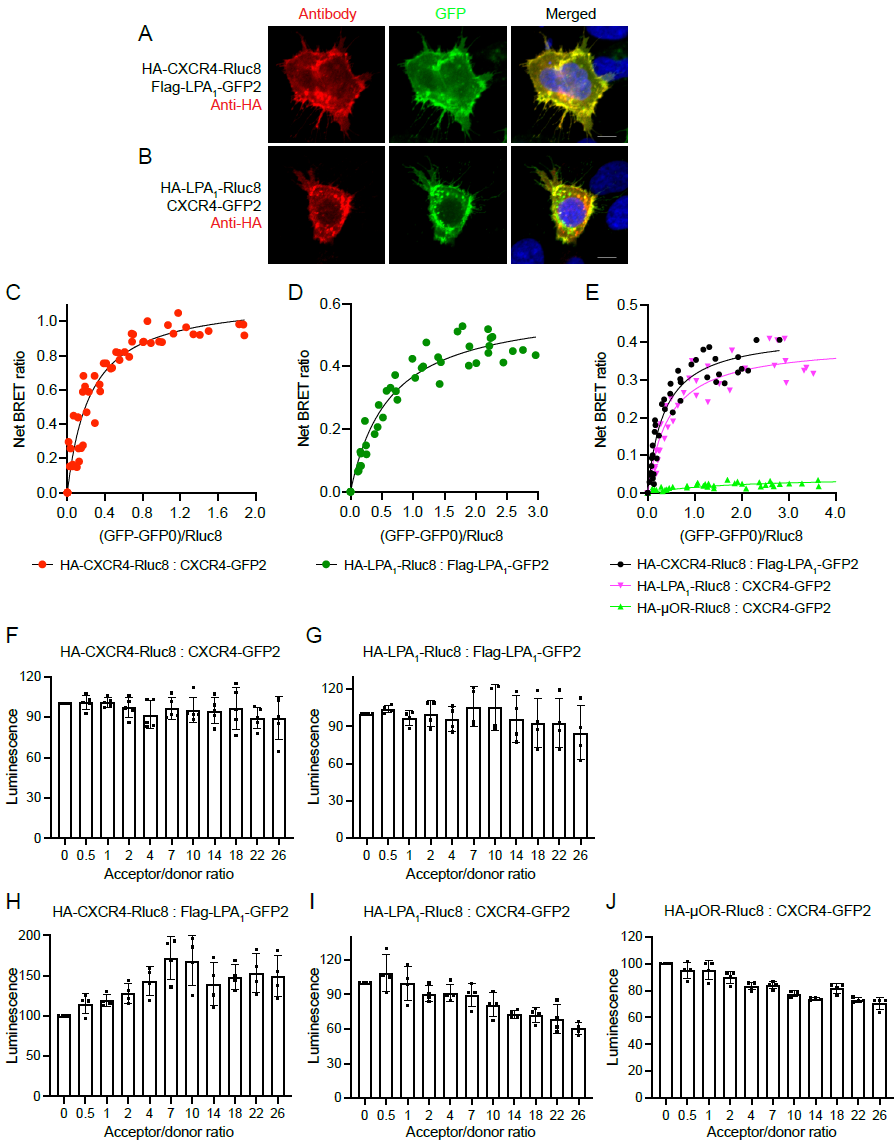


**Fig. S1.** BRET saturation assay performed in HEK293A cells. (A, B) Representative immunofluorescence images to visualize the localization of CXCR4 and LPA_1_. Cell nuclei were stained with Hoechst 33342. Scale bars: 10 μm. (C-E) BRET saturation assay for homodimerization and heterodimerization of CXCR4 and LPA_1_. Results were analyzed by nonlinear regression on a pooled data set. Data from *n* = 4 to 5 independent experiments (performed in triplicate) are presented. (F-J) Relative luminescence intensities of Rluc8-tagged GPCRs (donor) were measured as the expression of GFP2-tagged GPCRs (acceptor) was increased. Data represent the mean ± SEM of *n* = 4 to 5 independent experiments performed in triplicate.


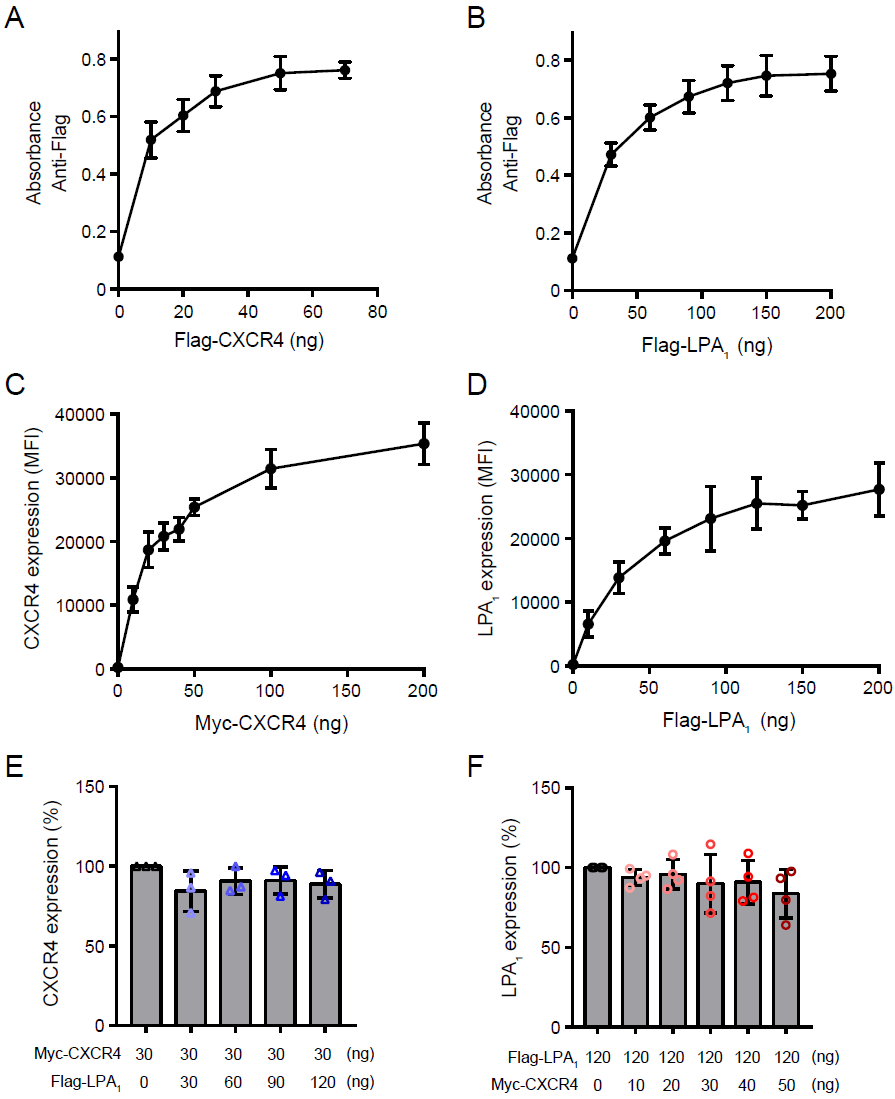


**Fig. S2.** Cell surface expression of CXCR4 and LPA_1_. (A, B) HEK293A cells were transfected with increasing amounts of Flag-CXCR4 (A) and Flag-LPA_1_ (B). Cell surface expression of Flag-CXCR4 and Flag-LPA_1_ was measured by ELISA using anti-Flag antibody without permeabilization. Data represent the mean absorbance ± SD of *n* = 3 independent experiments. (C) HEK293A cells were transfected with increasing amounts of Myc-CXCR4, and cell surface expression of Myc-CXCR4 was measured by flow cytometry using anti-Myc antibody. (D) HEK293A cells were transfected with increasing amounts of Flag-LPA_1_, and cell surface expression of Flag-LPA_1_ was measured by flow cytometry using anti-Flag antibody. Data represent the mean fluorescence intensity (MFI) value ± SD of *n* = 3 to 4 independent experiments. (E) Cell surface expression of Myc-CXCR4 was measured by flow cytometry in cells transfected with a fixed amount of Myc-CXCR4 (30 ng) and increasing amounts of Flag-LPA_1_ (0 to 120 ng). (F) Cell surface expression of Flag-LPA_1_ was measured by flow cytometry in cells transfected with a fixed amount of Flag-LPA_1_ (120 ng) and increasing amounts of Myc-CXCR4 (0 to 50 ng). Data represent the mean ± SD of *n* = 3 to 4 independent experiments.


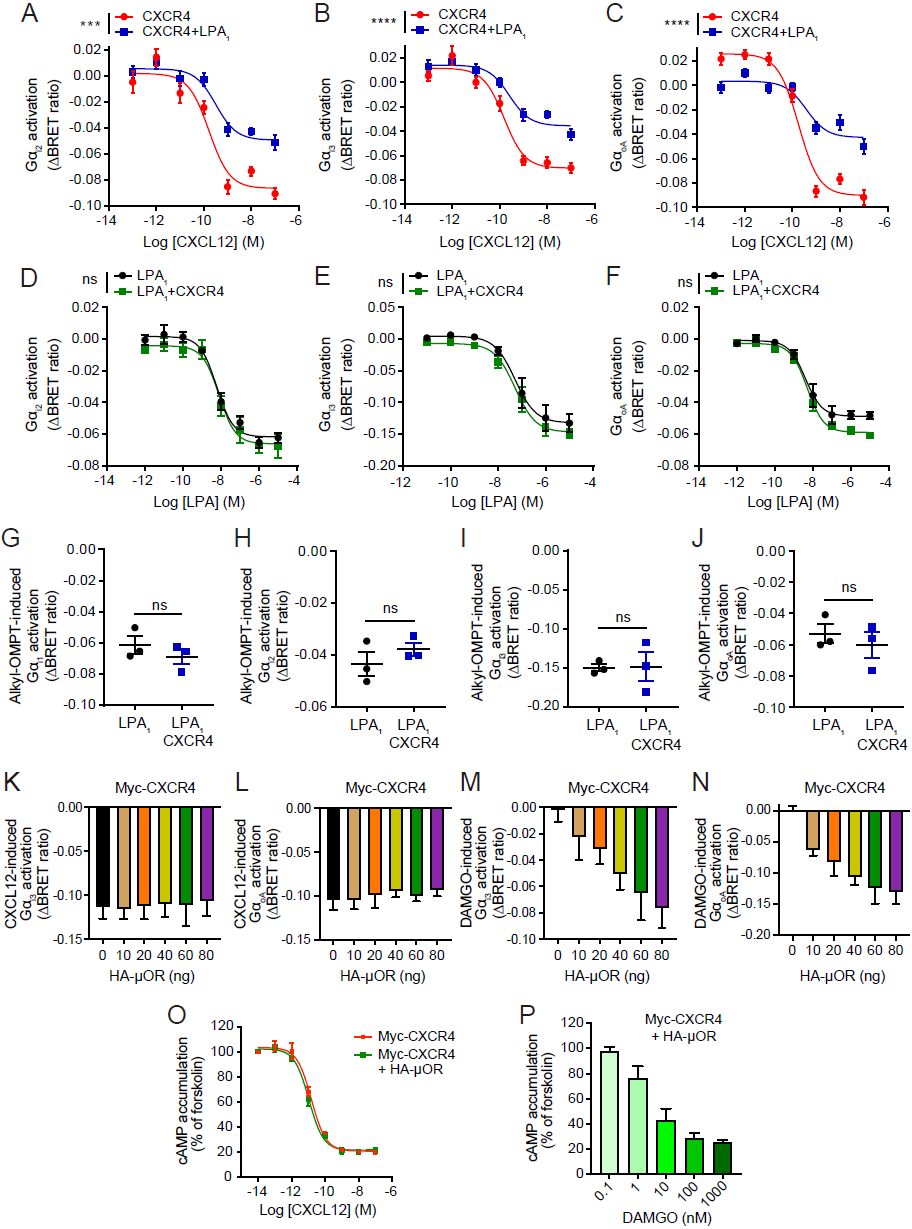


**Fig. S3.** Evaluation of heterotrimeric G protein activation. (A-C) HEK293A cells were transfected with Gα_i2_-Rluc8 (A), Gα_i3_-Rluc8 (B), or Gα_oA_-Rluc8 (C) in the presence of Gβ_1_, Gγ_1_-EYFP, and Myc-CXCR4 together with (Blue) or without Flag-LPA_1_ (Red). (D-F) HEK293A cells were transfected with Gα_i2_-Rluc8, Gβ_3_, and Gγ_8_-GFP2 (D), Gα_i3_-Rluc8, Gβ_3_, and Gγ_9_-GFP2 (E), or Gα_oA_-Rluc8, Gβ_3_, and Gγ_8_-GFP2 (F) in the presence of Flag-LPA_1_ together with (Green) or without Myc-CXCR4 (Black). (G-J) HEK293A cells were transfected with Gα_i1_-Rluc8, Gβ_3_, and Gγ_9_-GFP2 (G), Gα_i2_-Rluc8, Gβ_3_, and Gγ_8_-GFP2 (H), Gα_i3_-Rluc8, Gβ_3_, and Gγ_9_-GFP2 (I), or Gα_oA_-Rluc8, Gβ_3_, and Gγ_8_-GFP2 (J) in the presence of Flag-LPA_1_ together with (Blue) or without Myc-CXCR4 (Black). Data represent the mean ± SEM of *n* = 3 to 4 independent experiments (performed in triplicate). Statistical significance was tested using two-away ANOVA followed by Bonferroni’s multiple comparison test (A-F) or unpaired two-tailed Student’s *t* test (G-J). ****P* < 0.001; *****P* < 0.0001; ns, not significant. (K-N) CXCL12 (10 nM)-induced G protein activation (K, L) and DAMGO (1 μM)-induced G protein activation (M, N) were measured in HEK293A cells. (O, P) The effect of CXCL12 (30 nM) and DAMGO (1 μM) on forskolin (3 μM)-induced cAMP production was measured using a GloSensor cAMP assay. Data represent the mean ± SEM of *n* = 3 independent experiments (performed in triplicate).


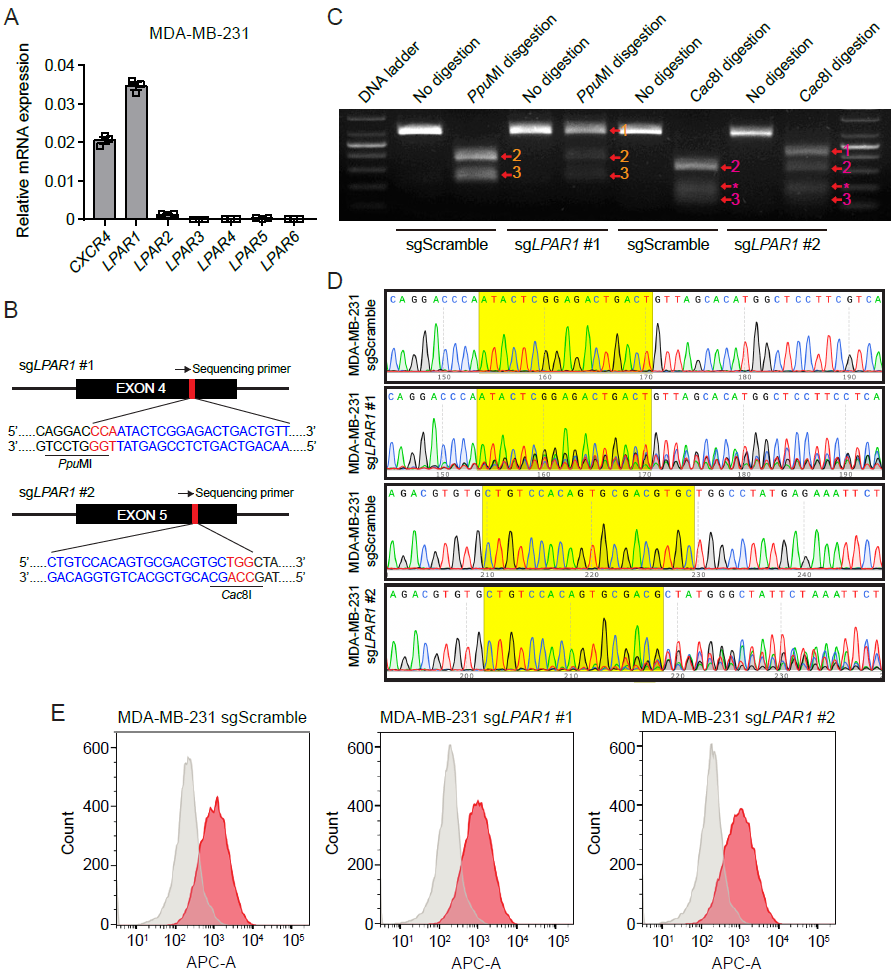


**Fig. S4.** Expression of GPCRs in MDA-MB-231 cells and validation of *LPAR1* knockout. (A) Gene expression levels of *CXCR4*, *LPAR1*, *LPAR2*, *LPAR3*, *LPAR4*, *LPAR5*, and *LPAR6* were measured in MDA-MB-231 cells. The relative expression level of each GPCR was normalized to that of GAPDH. Data represent the mean ± SEM of *n* = 3 independent experiments (performed in triplicate). (B) The diagram of the *LPAR1* gene showing the target guide RNA sequences (blue), the PAM sites (red), and restriction enzyme sites. (C) PCR-RE analysis of the PCR amplicons for sg*LPAR1* #1 and sg*LPAR1* #2. The predicted PCR amplicon sizes for sg*LPAR1* #1 and #2 are 587 bp and 584 bp, respectively. The first and last lanes are 100-bp DNA ladders. Arrow 1 indicates a fragment that was not cut by the restriction enzyme due to mutations around the PAM site. Arrow 2 and 3 indicate fragments that were formed by the restriction enzyme cutting at the PAM site of the PCR amplicon. Arrow * indicates a fragment that was formed by *Cac*8I cutting at the site other than the PAM site. (D) Sanger sequencing for the detection of targeted mutation around the PAM sites in the *LPAR1* locus. The target sequences are marked in yellow. (E) Cell surface expression of CXCR4 was analyzed by flow cytometry in MDA-MB-231 cells targeted with sgScramble, sg*LPAR1* #1, or sg*LPAR1* #2 using an isotype control (gray) and anti-CXCR4 antibody (red).


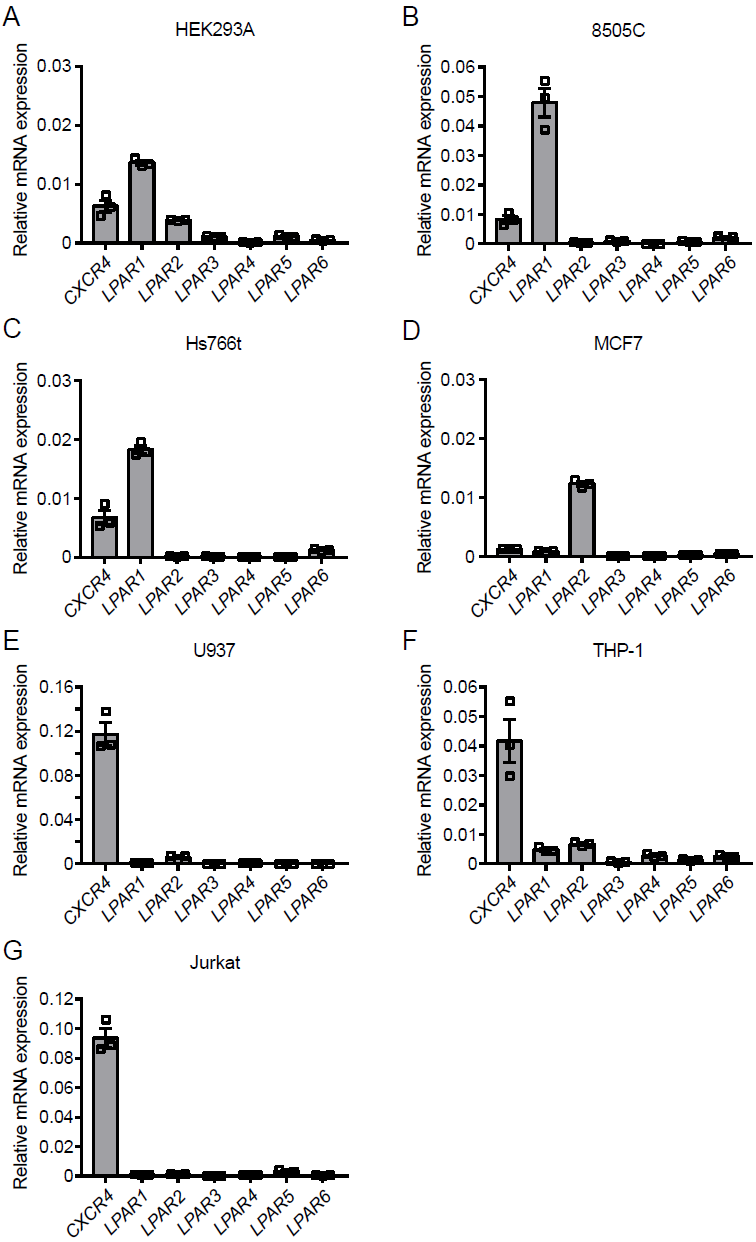


**Fig. S5.** Expression of CXCR4 and LPA receptors in various human cell lines. (A-G) Gene expression levels of *CXCR4*, *LPAR1*, *LPAR2*, *LPAR3*, *LPAR4*, *LPAR5*, and *LPAR6* were measured in HEK293A (A), 8505C (B), Hs766t (C), MCF7 (D), U937 (E), THP-1 (F), and Jurkat cells (G). The relative expression level of each GPCR was normalized to that of GAPDH. Data represent the mean ± SEM of *n* = 3 independent experiments (performed in triplicate).


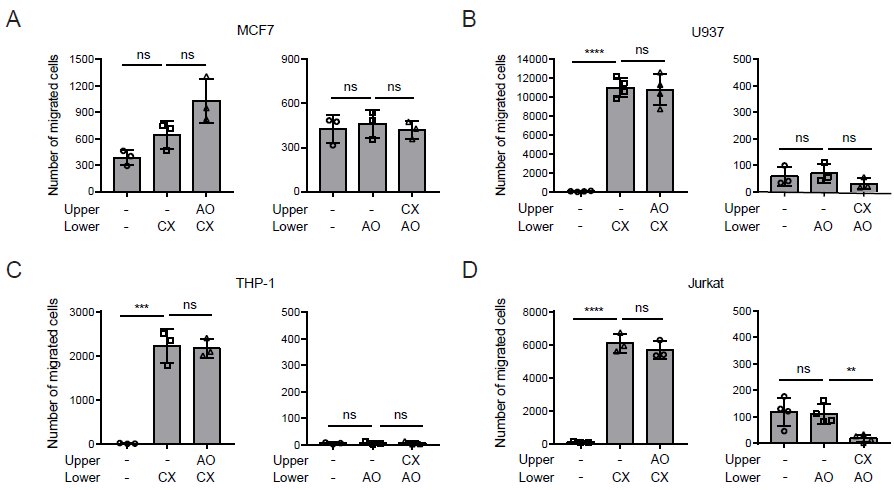


**Fig. S6.** CXCR4-mediated migration in cell lines that do not express LPA_1_. (A-D) The effect of alkyl-OMPT on CXCL12-induced cell migration was assessed using a transwell migration assay with alkyl-OMPT (1 μM) in the upper chamber and CXCL12 (10 nM) in the lower chamber (Left panels). The effect of CXCL12 on alkyl-OMPT-induced cell migration was assessed using a transwell migration assay with CXCL12 (10 nM) in the upper chamber and alkyl-OMPT (1 μM) in the lower chamber (Right panels). Migrated cells were counted from randomly selected images of 10 fields (A) or counted by flow cytometry (B-D). Data represent the mean ± SD of *n* = 3 to 4 independent experiments. Statistical significance was tested using unpaired two-tailed Student’s *t* test. ***P* < 0.01; ****P* < 0.001; *****P* < 0.0001; ns, not significant. AO: alkyl-OMPT; CX: CXCL12.


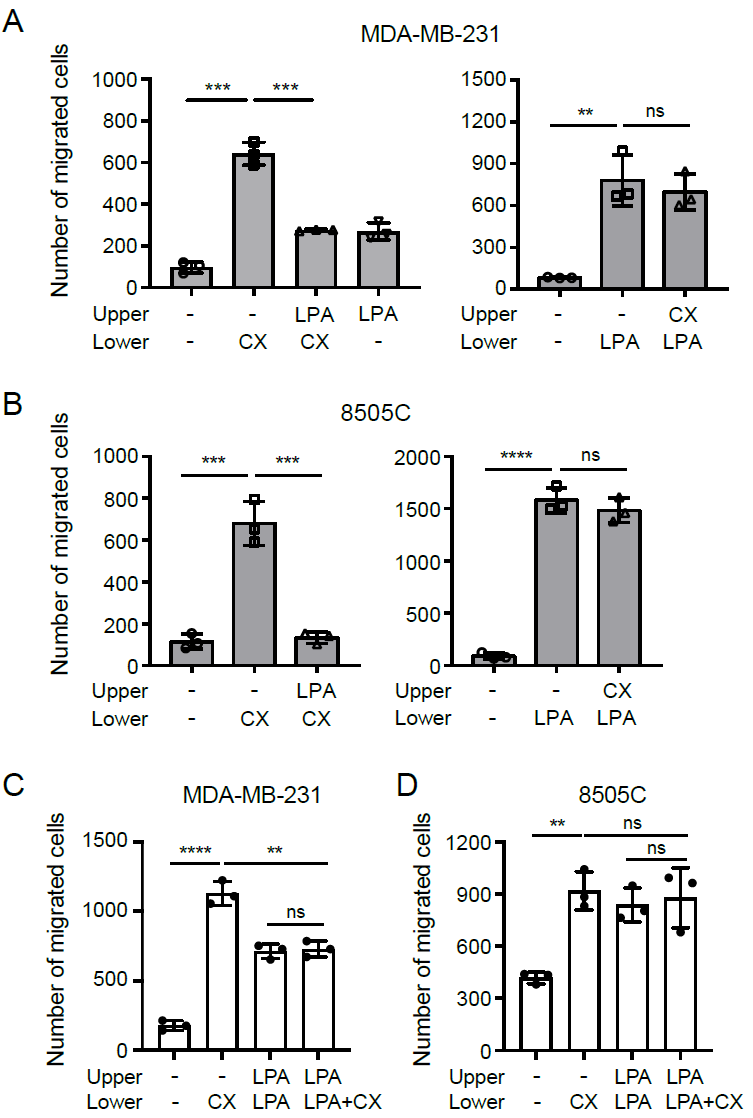


**Fig. S7.** The effect of LPA stimulation on CXCR4-mediated cell migration. (A, B) The effect of LPA stimulation on cell migration toward CXCL12 was assessed using a transwell migration assay with LPA (1 μM) in the upper chamber and CXCL12 (10 nM) in the lower chamber (Left panels). The effect of CXCL12 stimulation on cell migration toward LPA was assessed using a transwell migration assay with CXCL12 (10 nM) in the upper chamber and LPA (1 μM) in the lower chamber (Right panels). (C, D) The effect of LPA (1 μM) in both the upper and lower chambers on CXCL12 (10 nM)-induced cell migration was assessed in MDA-MB-231 (C) and 8505C cells (D). Migrated cells were counted from randomly selected images of 10 fields. Data represent the mean ± SD of *n* = 3 independent experiments. Statistical significance was tested using unpaired two-tailed Student’s *t* test. ***P* < 0.01; ****P* < 0.001; *****P* < 0.0001; ns, not significant. CX: CXCL12.


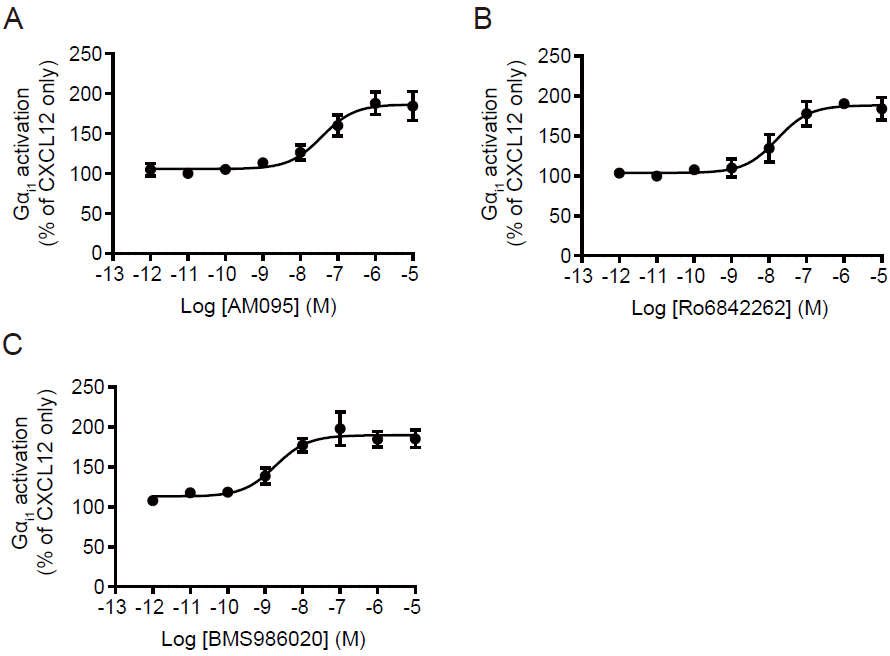


**Fig. S8.** The effect of LPA_1_ antagonists on CXCL12-induced G protein activity. (A-C) HEK293A cells were transfected with CXCR4 and LPA_1_ and pretreated with AM095 (A), Ro6842262 (B), or BMS986020 (C) at the indicated concentrations for 30 min. CXCL12 (10 nM)-induced Gα_i1_ activation was measured using BRET between Gα_i1_-Rluc8 and Gγ_9_-GFP2. Data represent the mean ± SEM of *n* = 3 independent experiments (performed in triplicate).


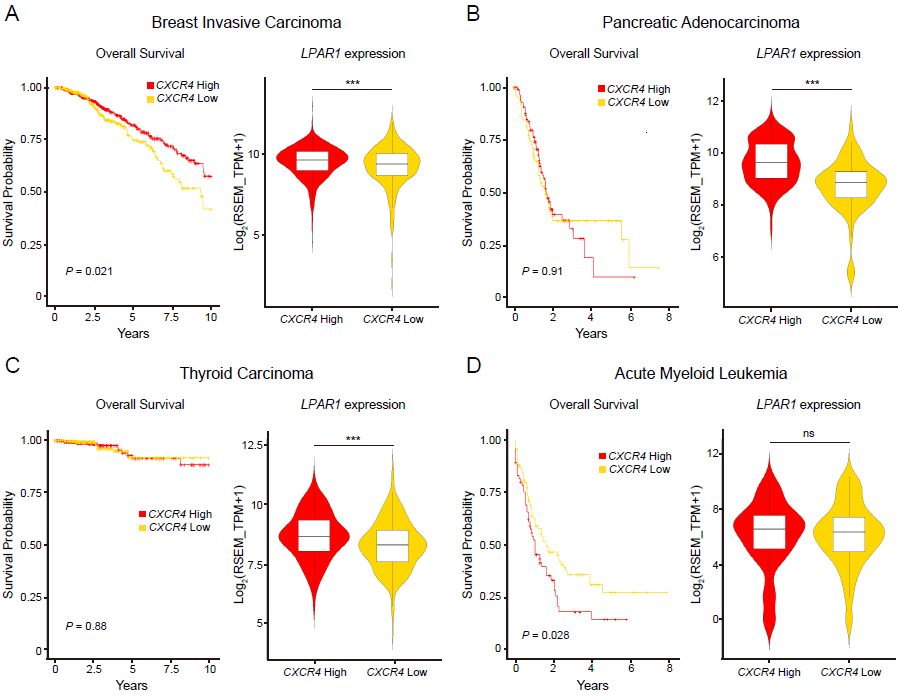


**Fig. S9.** Overall survival and expression analysis of *CXCR4* and *LPAR1* in the TCGA datasets. (A-D) The TCGA data for breast (A), pancreatic (B), thyroid cancers (C), and acute myeloid leukemia (D) were analyzed. (Left panels) OS for each cancer type was analyzed according to *CXCR4* expression level. Differences in OS between the “*CXCR4* high” and “*CXCR4* low” groups were compared using Kaplan-Meier curves, and *P* values were calculated with log-rank test using the Survival package in R. (Right panels) Each cancer type was divided by *CXCR4* expression level, and *LPAR1* expression between the “*CXCR4* high” and “*CXCR4* low” groups was compared. Statistical significance was tested using one-way ANOVA. ****P* < 0.001; ns, not significant.
